# Viral deep sequencing needs an adaptive approach: IRMA, the iterative refinement meta-assembler

**DOI:** 10.1186/s12864-016-3030-6

**Published:** 2016-09-05

**Authors:** Samuel S. Shepard, Sarah Meno, Justin Bahl, Malania M. Wilson, John Barnes, Elizabeth Neuhaus

**Affiliations:** 1 Influenza Division, Centers for Disease Control and Prevention, 1600 Clifton Road, Atlanta, GA 30329 USA; 2 Center for Infectious Diseases, The University of Texas School of Public Health, Houston, TX USA; 3 Battelle Memorial Research Institute, 1600 Clifton Road, Atlanta, GA 30329 USA

**Keywords:** Deep sequencing, NGS, Influenza, Ebola, Surveillance, High throughput, Public health

## Abstract

**Background:**

Deep sequencing makes it possible to observe low-frequency viral variants and sub-populations with greater accuracy and sensitivity than ever before. Existing platforms can be used to multiplex a large number of samples; however, analysis of the resulting data is complex and involves separating barcoded samples and various read manipulation processes ending in final assembly. Many assembly tools were designed with larger genomes and higher fidelity polymerases in mind and do not perform well with reads derived from highly variable viral genomes. Reference-based assemblers may leave gaps in viral assemblies while *de novo* assemblers may struggle to assemble unique genomes.

**Results:**

The IRMA (iterative refinement meta-assembler) pipeline solves the problem of viral variation by the iterative optimization of read gathering and assembly. As with all reference-based assembly, reads are included in assembly when they match consensus template sets; however, IRMA provides for on-the-fly reference editing, correction, and optional elongation without the need for additional reference selection. This increases both read depth and breadth. IRMA also focuses on quality control, error correction, *indel* reporting, variant calling and variant phasing. In fact, IRMA’s ability to detect and phase minor variants is one of its most distinguishing features. We have built modules for influenza and ebolavirus. We demonstrate usage and provide calibration data from mixture experiments. Methods for variant calling, phasing, and error estimation/correction have been redesigned to meet the needs of viral genomic sequencing.

**Conclusion:**

IRMA provides a robust next-generation sequencing assembly solution that is adapted to the needs and characteristics of viral genomes. The software solves issues related to the genetic diversity of viruses while providing customized variant calling, phasing, and quality control. IRMA is freely available for non-commercial use on Linux and Mac OS X and has been parallelized for high-throughput computing.

**Electronic supplementary material:**

The online version of this article (doi:10.1186/s12864-016-3030-6) contains supplementary material, which is available to authorized users.

## Background

Influenza viruses cause a significant disease burden as a result of seasonal activity and outbreaks. During the 2012–2013 influenza season in the United States, influenza virus caused an estimated 633,001 hospitalizations including 27,810 deaths [1]. Due to the potential severity of this disease and the fast mutation rate of the virus, a large global surveillance network is required to monitor circulating strains in order to characterize variants and select regional vaccines. In 2014, over 300,000 influenza samples were collected in 124 countries [2] and global surveillance efforts deposited over 80,000 gene segments in GenBank and GISAID. At the Centers for Disease Control and Prevention (CDC) in Atlanta, Georgia, USA, over 6500 influenza gene segments were sequenced in 2014. About 1 out of every 4 of these segments was generated using next generation sequencing (NGS) technology. In 2015, almost all gene segments were sequenced using NGS (93 % of 23,000 generated). Next generation sequencing of influenza viruses, compared to traditional methods (such as Sanger sequencing), increases sample multiplexing by up to 48 fold and coverage depths by up to 1000 fold. These benefits allow for greater overall throughput and increased accuracy in studying sub-populations within viral samples; however, keeping up with the NGS data deluge is challenging, particularly for influenza viruses.

Influenza is an RNA virus that packages eight separate gene segments, including an error-prone polymerase complex, into each glycoprotein-coated virion. These genes segments code for hemagglutinin (HA), neuraminidase (NA), matrix protein (M1), ion-channel protein (M2), nucleoprotein (NP), non-structural protein (NS) and three polymerase proteins (P1, PB1, and PB2). The polymerase complex typically generates one spontaneous mutation during each replication cycle [3], thereby accounting for a mean substitution rate of 10^−3^ substitutions per site per year [3]. Moreover, a single host that has been simultaneously infected with two or more different virus strains can serve as a mixing vessel in which novel virions are formed. This process, known as virus reassortment, can cause outbreaks of infection, with potentially dire consequences, if the new virus is both pathogenic and readily transmitted.

The high level of mutation inherent in influenza virus reproduction leads to antigenic drift within gene segments while reassortment of segments causes antigenic shift [4]. Antigenic drift and shift events create virus strains that may not be efficiently captured in host immune responses generated by current vaccines and it is therefore important to track changes in all gene segments during surveillance of circulating influenza virus strains. Until recently, only HA, NA and M gene segment changes of circulating human seasonal influenza virus strains were routinely tracked, but the emergence and detection of highly pathogenic avian influenza strains caused a rapid expansion in surveillance efforts for zoonotic viruses to the extent that sequencing of all gene segments of avian and swine viruses is now typical and routine. Likewise, using NGS technologies, it is now not only feasible but also routine to sequence all gene segments of the comparatively large number of sampled seasonal human influenza viruses.

Even though it is becoming easier to use NGS methods to generate whole genome sequence data for influenza virus surveillance, the diversity and mutation rate of influenza, like other RNA viruses, presents a challenge to high-throughput NGS assembly efforts. Typical reference-based NGS assembly programs were written for eukaryotic and prokaryotic organisms with non-segmented genomes [5–8] that undergo slower mutation rates than RNA viruses. These programs discard read sequences from assembly that have too many mismatches or insertions/deletions *(indels*) versus a defined reference sequence within some configurable scoring threshold. Read sequences containing variants are more frequently discarded from reference-based assemblies due to an overall mismatch to the reference and it is possible, therefore, for thousands of acceptable reads to be excluded, thereby minimizing overall coverage and preventing complete assembly. Because RNA viruses may contain phased minor variants at almost any frequency or position, a unique approach is required to efficiently assemble NGS virus reads while maximizing overall coverage, even when high coverage depths are achieved.

**Fig. 1:**
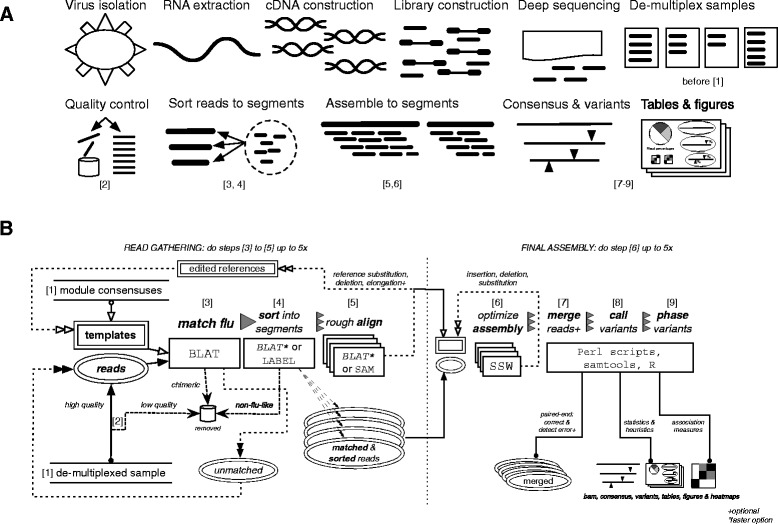
Iterative refinement meta-assembler (IRMA) workflow: the influenza module. (**a**) The general process of sequencing a segmented RNA virus and assembling with IRMA. (**b**) Diagram of IRMA steps 1 through 9, showing the iterative processes involved. Steps in (**b**) are also labeled under the steps of (**a**) where they correspond

Reference-based assembly methods do not perform universally well for segmented viruses that rapidly evolve and re-assort. Therefore, we developed a flexible approach that more thoroughly addresses viral diversity. Here we introduce IRMA, the iterative refinement meta-assembler (see Fig. 1). IRMA is routinely used to process genome sequence data derived from the large volume of surveillance specimens characterized at CDC. While previous solutions have addressed specific challenges such as quality control [5], variant calling [6, 7], phasing [8], and assembly [9], IRMA provides a comprehensive solution to address each aspect of NGS assembly, as it applies to RNA virus evolution, in a flexible and robust manner and has been used successfully to identify low frequency *indel* variants in ebolavirus populations [10].

Our software is *not* another new assembler; rather, it is a *meta-*assembler that ties together and customizes components and options we have found relevant to the viral genetic landscape. IRMA provides segment level read sorting based on LABEL, a sequence classification tool ideal for segmented genomes [11]; iteratively gathers reads and iteratively edits the reference templates to account for high population diversity and mutational rates; and provides redesigned variant calling (heuristics as well as statistical tests) and phasing to allow for the analysis of diverse viral sub-populations. IRMA automates quality control, such as the removal of short, low quality, and chimeric reads, and gives options for reference elongation to discover novel untranslated regions (UTRs). In order to handle the ever growing demand for high volume sequence assembly and analysis, IRMA parallelizes tasks at almost every stage—by single multi-core computer or by grid—and integrates different tools and options to let the user determine the appropriate burden of compute time versus accuracy. Named configuration files are used to adapt assembly options to the strengths of particular deep sequencing platforms while ensuring ease of use and consistency. In the future we plan to integrate new tools (such as Bowtie2), features, and create ready-to-go modules for viruses beyond influenza and ebola.

## Results & discussion

Deep sequencing technology makes it possible to go beyond the generation of simple consensus sequences. One can estimate the frequencies of both consensus and minority alleles with greater confidence as coverage depth increases. These estimations can be helpful in studying viral adaptation, testing for low-level virulence markers, and measuring overall viral diversity within a sample. Unfortunately, it is difficult to know how far calculations differ from reality and how much assembly methodology can impact these estimation calculations.

Public health surveillance of viral pathogens requires high throughput assembly of varied and novel genetic variants. For reference-based assembly strategies, a close reference set will help capture and identify appropriate reads. However, an appropriate reference may not exist (one does not exist in the public record), or, may be cumbersome to provide (i.e., a representative sequence from the same clade was missing from the standard reference dataset). Determining an appropriate reference set for viruses like influenza is further complicated by fast mutation rates and the capability to reassort. Influenza viruses vary approximately 10^−3^ nucleotides/site/year, and yet individual gene segments may have differing internal mutation rates as well as independent evolutionary histories [3].

We solve these referenced-based assembly complications by using iterative refinement—moving the reference template closer to the reads—to obtain quality assemblies with increased sensitivity to distant and novel genetic variants. The concept of iterative refinement has been exploited for many years for multiple sequence alignment purposes [12] and works well for optimizing the number of reads included for final assembly genomes as well in the assembly process itself. Iterative refinement has also independently been applied to viral *de novo* assemblers to improve performance. Unfortunately, and unlike reference-based assembly, state-of-the-art *de novo* assemblers produce unique influenza genomes only 21 % of the time [13], making them poor candidates for the high throughput NGS surveillance of RNA viruses.

The general process of sequencing a segmented RNA virus and the implementation, at a high level, of IRMA as used for surveillance is shown in Fig. 1a. Figure 1b provides details of the IRMA pipeline, showing the iterative processes involved. The first phase (steps 1–5) of the IRMA pipeline focuses on gathering as many NGS reads as possible by matching them to reference sequences, sorting them into their respective gene segments, and using the results of a rough alignment to edit the references. Any unmatched reads go back into the pool for additional rounds of matching after the references are refined. It is this step that ensures unmatched reads are not unnecessarily discarded because of the use of genetically distant reference sequences. The second phase (steps 6–9) of the IRMA pipeline finalizes the assemblies by iteratively editing references in order to find an optimal assembly score, after which read-pairs may be merged, statistics tabulated, variants called, and figures drawn. See Methods section 3 or section Iterative refinement meta-assembler algorithm (Fig. 1b) for full details.

We have successfully used IRMA on surveillance data of over a dozen different influenza A subtypes as well as on both influenza B lineages. We have tested IRMA on PacBio, Ion Torrent, and the Illumina MiSeq platform; however, for this study we have used the MiSeq’s paired-end read technology for our sequencing experiments (see Sequencing and mixture protocol and Sequencing and random priming protocol) to emphasize our read-pair merging (section Reference based read-pair merging and correction (Fig. 1b, step 7))*,* read-pair overlap error estimation (section Variant calling (Fig. 1b, step 8))*,* and short-read phasing solutions (section Qualitative phasing of linked variants (Fig. 1b, step 9)). For this study we used H3N2 human seasonal data for our viral mixture experiments (Tables 1 and 2), where tracking minor variants over time is of increasing interest to surveillance programs. We used H7N7 as a representative of avian subtypes, where novel variants might be expected to appear periodically, to demonstrate the importance of IRMA’s iterative refinement methodology for a smaller subset of non-human data.

**Table 1:** Average absolute error and standard deviation (parentheses) are shown for each group & parameterization.

				Allele type	
Reference	Program	QC	Observations	Consensus	Minority
Flu gene segment consensuses	Bowtie2	**-**	1.73Gb	0.30 % (2.97 %)	0.15 % (2.22 %)
		**+**	1.61Gb	0.28 % (2.86 %)	0.15 % (2.36 %)
	IRMA	**-**	1.99Gb	0.17 % (0.47 %)	0.08 % (0.29 %)
		**+**	1.97Gb	0.12 % (0.42 %)	0.05 % (0.27 %)
Assembly controls: both unmixed genomes	IRMA^*^	**+**	1.97Gb	0.12 % (0.40 %)	0.05 % (0.26 %)
	Bowtie2	**+**	1.97Gb	0.12 % (0.40 %)	0.05 % (0.26 %)

Observations are the total number of assembled nucleotides for all genes and mixtures in triplicate. Allele type is per mixture-replicate-gene-position showing consensus (99.994% majority, 0.006% plurality) allele error and minority allele (non-consensus) error. Bowtie2 was set to very sensitive, local assembly.

*IRMA with a single round of read gathering iterations, final assembly using original references

**Table 2:** Significance testing of variant alleles on H3 influenza mixtures

	Assembly-specific error test				Allele-specific error test				Assembly + Allele tests			
Mix-in	Major change		Negligible		Major change		Negligible		Major change		Negligible	
Percent	Fails	Sig.	Fails	Sig.	Fails	Sig.	Fails	Sig.	Fails	Sig.	Fails	Sig.
0 %	99.9 %	0.1 %	99.9 %	0.1 %	98.2 %	1.8 %	99.6 %	0.4 %	100 %		99.9 %	0.1 %
0.5	70.2	29.8	99.9	0.1	9.8	90.2	99.7	0.3	70.2	29.8	100	0.0
1	3.5	96.5	99.9	0.1		100	99.6	0.4	3.5	96.5	99.9	0.1
2	0.2	99.8	99.9	0.1		100	99.6	0.4	0.2	99.8	99.9	0.1
5		100	99.9	0.1		100	99.6	0.4		100	99.9	0.1
10		100	99.9	0.1		100	99.6	0.4		100	99.9	0.1
20		100	99.9	0.1		100	99.5	0.5		100	99.9	0.1
25		100	99.9	0.1		100	99.6	0.4		100	100	0.0
50		100	99.9	0.1		100	99.5	0.5		100	99.9	0.1

Negligible allele mixtures meant both unmixed parent viruses had frequencies ≥ 98 % or ≤ 1 % while major allele mixtures were defined as one pre-mixture donor virus having ≥ 98 % frequency and the other ≤ 1 % frequency. Variant negatives (negligible mixtures or 0 % mix-in) are highlighted in yellow while variant positives are in green. Cell data were omitted when counts were zero. The null hypothesis was that variants were produced by sequencer error. All tests were with respect to second-order corrected, one-sided 99.9 % binomial confidence intervals. The percentages of minor variant alleles not distinguishable from sequencer error is marked “fails” for failing to reject the null hypothesis. The percentage of variants rejecting the null hypothesis is marked “sig.” for significant and are candidates for calling single nucleotide variants

### Influenza genetic diversity is too varied for majority consensus to capture

**Fig. 2:**
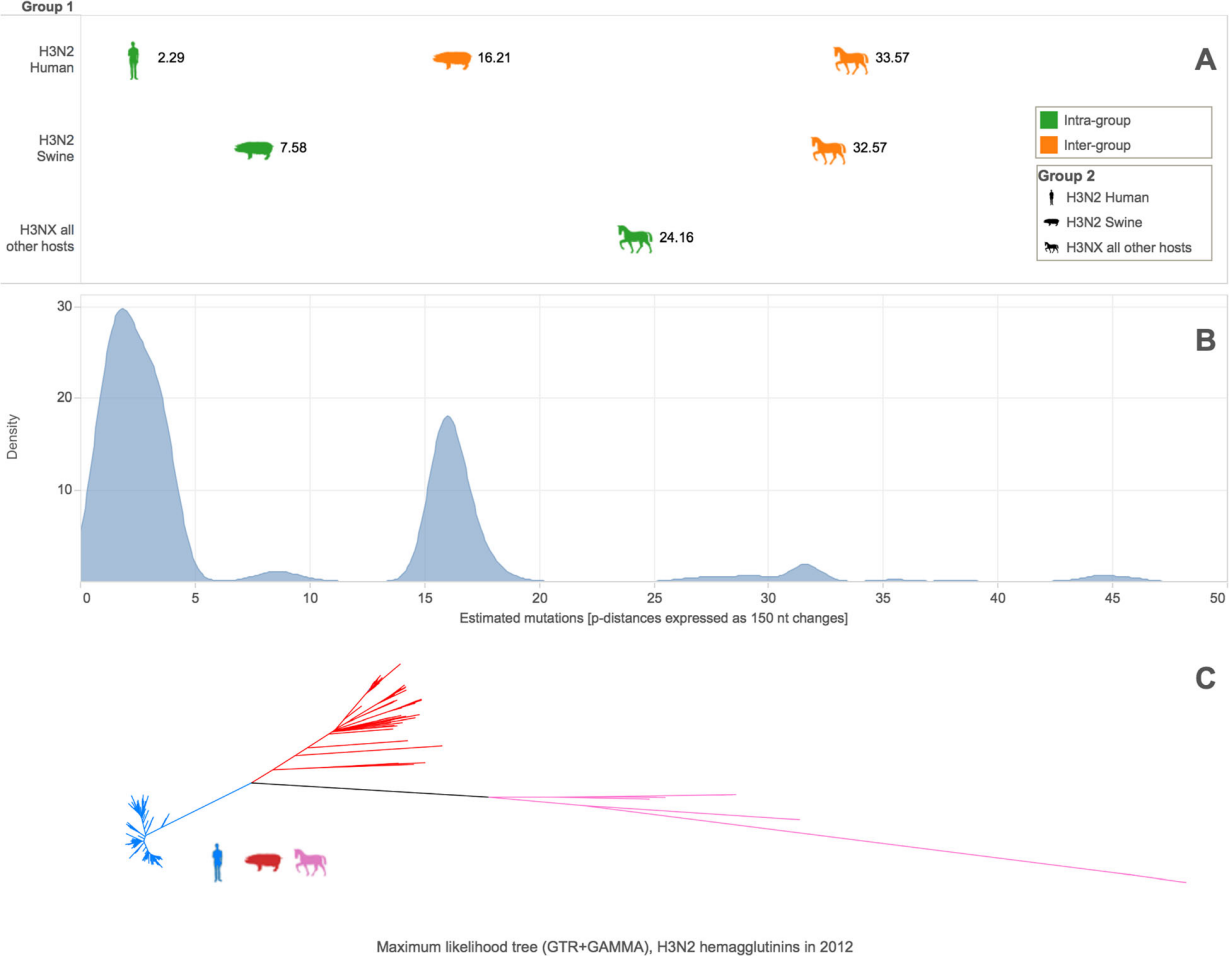
Genetic diversity of 1097 influenza A(H3) hemagglutinins collected in 2012. **a** Upper triangle of the host group-to-group average pairwise distance matrix plotted on and expressed as the number of estimated mutations per 150 nucleotides. **b** Density plot for the upper triangle of the pairwise distance matrix over all sequences, plotted and expressed on the same scale. **c** Maximum likelihood phylogenetic tree of H3 *HA* sequences with labeling by host group color

Influenza genetic diversity is vast. To illustrate this diversity, we combined sequence data for 1097 influenza A(H3) viruses collected in 2012 and assessed differences amongst members of the set. Viruses of this subtype may circulate in humans, swine, horses, dogs, and other animals. Figure 2a shows that host groups (human, swine, or other) are, on average, 16, 32, or 33 mutations apart (per 150 nucleotides) while variation within a host group is quite appreciable (2, 7, or 24 mutations respectively). Figure 2b shows the pairwise sequence distances as a density plot, revealing that the shape of the viral genetic landscape of one gene from one HA subtype isolated in 1 year is multi-modal and complex. Figure 2c supports this observation with a maximum likelihood phylogenetic tree.

**Fig. 3:**
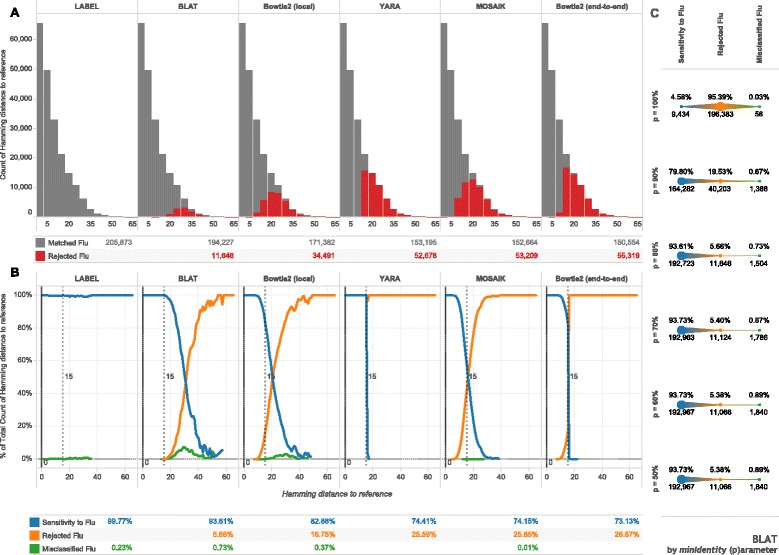
Sensitivity to influenza biological diversity. For each influenza type, subtype, and gene segment—39 alignments in all—randomly chosen subsequences of fixed length (150 nucleotides) were matched against alignment consensuses with programs shown. Hamming distance is the number of mismatching nucleotides between subsequences and references. **a** Histograms, with binning width 5, give the count of the subsequences matched to a flu consensus sequence or not. **b** Line plots show normalized frequencies at each un-binned hamming distance and further characterize matched fragments into the proportion of misclassified fragments (matched to the wrong influenza consensus). Tabular summaries represent the total count or proportion for each method across all 205,873 subsequences. The dashed vertical lines represent the general limit of detection for non-statistical approaches. **c** The minimum identity parameter (*p)* for BLAT was varied on the same dataset with summary proportions and counts shown for each parameter value

Such genetic diversity can overwhelm standard match & align approaches, as can be seen by comparing the results of several different alignment strategies on short sequence samples from our full influenza reference dataset (section Influenza alignment dataset) versus a set of consensus references. We selected BLAT, Bowtie2, and MOSAIK as representatives of local alignment strategies, used YARA and Bowtie2 (end-to-end mode) as an example of semi-global alignment, and LABEL for machine learning and statistical approaches (see section Random subsequence datasets (Fig. 3 & Additional file 1)). Figure 3a illustrates that a 10 % difference from the assembly reference (15/150nts) is enough to start losing sensitivity for local aligners, and by design, for semi-global aligners. LABEL (used to sort reads into segment bins in the IRMA pipeline) uses profile hidden Markov models along with a support vector machine for classification of reads instead of capturing them based on overall match to a reference set [11]. Capturing reads using a statistical model is expected to be more sensitive to actual influenza diversity. On the other hand, using a reference sequence is much more computationally expedient. Figure 3b shows how read misclassification (matching to influenza, but the wrong subtype or gene segment) can also occur for more sensitive approaches. This is consistent with the well-known [14] trade-off between sensitivity and specificity that can hamper the accuracy of more permissive classifiers. For example, our attempts to increase BLAT’s sensitivity by relaxing read matching criteria (Fig. 3c) provided diminishing returns (0.12 % more matches from 80 to 60 % minimum identity) while increasing the rate of flu read misclassification.

Mutations introduced during the sequencing process are difficult to distinguish from real, genetic variability. One can attempt to do so using accurate estimates of sequencer error, as discussed in later sections. However, the disconcerting issue is that genetic variability is compounded with the variability introduced by sequencing error, making the capture of distant reads even more difficult for RNA virus assembly. Given the reality of sequencing error and LABEL’s use of genetic diversity to train its algorithm, we reassessed the four best method’s sensitivity and classification accuracy on influenza data with artificially mutated sample subsequences from a set of references at various fixed distances (see section Influenza A(H3N2) genetic diversity, (Fig. 2)). Additional file 1 shows that sensitivity begins to drop after a 7 to 17 % difference from the reference set (depending on method). LABEL sensitivity is lower for artificial variation than for genetic variation (Fig. 3), due to its reliance on a training set of actual influenza sequences. Misclassification appears at 25 nucleotide differences (of 150nts) for most methods, except for YARA, which has a maximum edit distance. As an alternative LABEL strategy, the LABEL “trio” set of modules succeeds in reducing misclassification by focusing on classifying HA, NA and the internal gene segments separately (see section Influenza A(H3N2) genetic diversity, (Fig. 2) as well as Additional file 2). We therefore have chosen to use the LABEL “trio” module set in IRMA’s sorting step (Fig. 1b, step 4) to balance sensitivity & accuracy.

For reference-based strategies, a close reference set will help capture and identify appropriate reads, but such a dataset loses usefulness when the target sequence is unknown. Determining an appropriate reference set is complicated by influenza’s fast mutation rate—approximately 10^−3^ nucleotides/site/year—and reassortment, where individual gene segments may have independent evolutionary histories [3]. These complexities can be made more challenging by the addition of artificial variation, since only 10 % dissimilarity to reference is enough to start losing coverage depth.

### Iterative refinement increases sensitivity to distant genetic variants

**Fig. 4:**
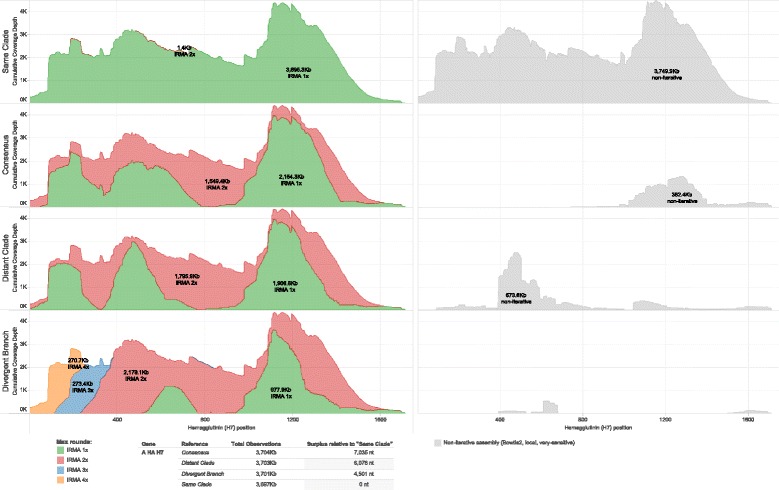
Heamgglutinin cumulative coverage depth by IRMA maximum (read-gather) rounds. A randomly primed A/equine/Detroit/3/64-like sample is assembled against influenza A H7 *HA* global consensus, and three other full CDS references from the H7 tree using IRMA with max rounds set to 1, 2, 3 and 4. Non-iterative assembly (Bowtie2, local, very-sensitive) to the same references is in *light gray*

**Fig. 5:**
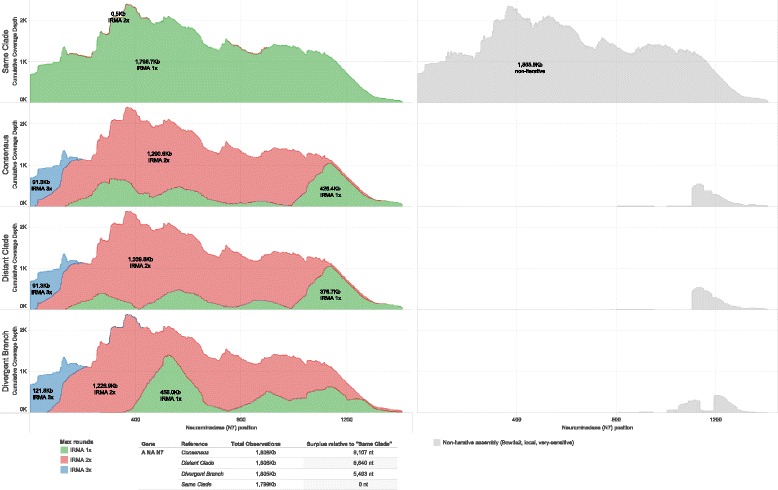
Neuraminadase cumulative coverage depth by IRMA maximum (read-gather) rounds. A randomly primed A/equine/Detroit/3/64-like sample is assembled against influenza A N7 *NA* global consensus, and three other full CDS references from the N7 tree using IRMA with max rounds set to 1, 2, 3 and 4. Non-iterative assembly (Bowtie2, local, very-sensitive) to the same references is in *light gray*

**Fig. 6:**
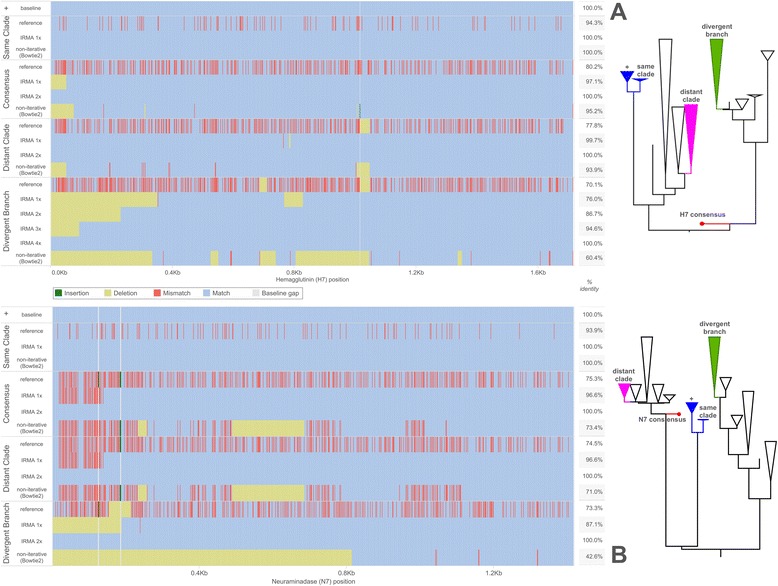
Assembled consensus differences to baseline. Known baseline consensus sequences correspond to an A/equine/Detroit/3/64-like sample. Differences versus the baselines are shown for (**a**) H7 *HA* and (**b**) N7 *NA* starting references and for the progression of assembled consensus sequences corresponding to each maximum IRMA round. A non-iterative assembly (Bowtie2, local, very sensitive) using each starting reference is shown for comparison. Match, mismatch, deletion, and insertion states are relative to the baseline in blue, red, yellow, and green respectively while white is used for a baseline gap in the alignment created by non-baseline insertions. Percent identity versus the baseline sequence is shown to the right of each graphed sequence. The phylogenetic trees depict approximate placement of the starting references on our H7 and N7 datasets, with the baseline labeled as “+”

Iteratively gathering reads (depicted in Fig. 1b, steps 3 to 5) for the assembly of real influenza virus A(H7N7) hemagglutinin (Fig. 4) and neuraminidase (Fig. 5) gene segments increases both coverage depth and breadth irrespective of starting consensus compared to a non-iterative approach. Figure 6 further shows the differences between the known baseline sequences and the final assembled consensuses. Phylogenetic trees are given to show the relationship between the starting references and the known baselines for each gene segment. We observe that, given enough rounds of gathering reads, IRMA can converge to the correct consensus sequence regardless of the starting reference used while running a standard assembly program non-iteratively (such as Bowtie2) yields a reference dependent result.

We believe IRMA is successful in assembling reads to more distant references because viral gene segments have varying levels of conservation across their length that allow for the capture of a portion of the reads. Furthermore, the first reads gathered will not, on average, solely overlap the more conserved regions but will span some of the less conserved regions as well. Combined with a slower but more sensitive statistical alignment strategy (SAM), reads can be accurately placed. Editing the reference set to the consensus of the data actually found moves the references closer to the read pool and allows for gathering even more distant reads.

### Using mixtures to calibrate assembly accuracy

Assembly error rates may vary from kit to kit, platform to platform [15], and run to run [16]. Base call quality scores, developed by manufacturers, attempt to account for the sequencer portion of these fluctuations, but they themselves are sometimes imperfect [17]. Whether or not all sources of error related to the sequencing process can be accurately estimated, the assembly portion should strive to do no harm. A big advantage of deep sequencing is that it provides for the detection of very low frequency minority alleles. Therefore, it is important to objectively assess whether allele frequencies are accurately estimated for any new NGS methodology.

In order to calibrate IRMA’s frequency calculations, we artificially mixed two closely related influenza A(H3N2) viruses in twn ratios, including two unmixed controls, in triplicate (see Sequencing and mixture protocol). The material was normalized and mixed post PCR amplification in order to correctly control for the amount of each virus added to the mixture. Proper assembly minimizes the absolute error from expected frequency (see Calibration calculations) and maximizes reproducibility (lowers standard deviations).

For an assembly control (Table 1), we assembled against both unmixed parent donor viruses. Such references will not be available in a real-world scenario without a priori knowledge (as here) or some kind of iterative refinement strategy. The results for the IRMA control (run non-iteratively) and Bowtie2 control (our representative of a standard approach) differ very little. However, when the reference sequences are less perfect—in this case our flu consensus set—the difference between IRMA and a non-iterative approach becomes apparent. IRMA assembled 15 & 22 % (depending on QC) more nucleotides than Bowtie2 (Table 1), reduced average absolute error by half, and lowered the standard deviation of the absolute error by over 6 fold. *Importantly, the relatively high variability (~2 % error) in the absolute error for the non-iterative approach (Bowtie2) means one cannot accurately characterize low frequency minority variants without finding a better reference first*. Fortunately, this is precisely what IRMA’s iterative process does for the user: it provides consistent, accurate results on par with assembly to a known, fixed reference without the need for reference management or highly homologous reference sequences. These features are important to the ongoing success of NGS on segmented RNA viruses (with high mutations rates and possible reassortment), regardless of the platform being used for sequencing.

Additionally, we note that the common practice of quality control (filtering by read length and median quality) reduces error for all methods (depicted in Fig. 1b, step 2). Read-pair merging, LABEL sorting, and alternative reference generation further reduce error by a small amount; conversely, the choice of Smith-Waterman parameters (weights) seems very important for proper assembly (Additional file 3). Alternative reference generation in addition to iterative refinement (see section Iterative final assembly (Fig. 1b, step 6)) produces an error profile most similar to controls (best possible assembly outcomes). Bowtie2 assembly to the consensus reference set occasionally lacked coverage breadth, but was never less than 93 % for any given gene segment (data not shown). These in-depth results, including relative error calculations, are given in Additional file 3. While IRMA is at least as good as and often better at accurately estimating allele frequencies than non-iterative approaches like Bowtie2, it is important to note that IRMA provides a viable solution for assembly when a reasonably homologous reference genome is not available. IRMA does not require post-process stitching together of *de novo* and referenced-based contigs, but rather, sorts short reads, refines the reference set and optimizes assembly without the need for manual reference management.

### Significance testing variants on influenza mixtures

Variant calling programs have been shown to produce diverse results for eukaryotic datasets [18]. However, to deal with the high mutation rate of RNA viruses, where variation can occur almost anywhere, we adopted a strategy that integrates both empirically derived heuristics and statistics. Heuristics can be customized to the organism and wet lab preparation at hand and can be adjusted to help capture non-sequencer sources of error. On the other hand, we based our statistical tests solely on multiple estimates of sequencer error—both assembly-specific and allele-specific measures. Such estimates of error must be shown to be reliable in the first place in order to be useful in variant calling. We therefore evaluated how accurate significance testing variants and non-variants would be on 99.9 % confidence intervals (see section Variant calling (Fig. 1b, step 8)). Since sequencer error estimates were used, the null hypothesis was that single nucleotide variants were generated by sequencer error. Table 2 shows the results for each significance test separately and together (rejecting both hypotheses) on a group of presumed real (green) and spurious (yellow) minor variants.

In a perfect scenario, where sequencer error rates are known and data are always binomially distributed, spurious variant allele frequencies will be contained within our confidence intervals 99.9 % of the time and will test significantly 0.1 % of the time. Remarkably, this is exactly the case for the error-generated variants (yellow, Table 2) versus our assembly-specific error tests. The reason: deep sequencing provides huge sample sizes to powerfully estimate statistics. For the 240 gene segment assemblies in our experiment, the number of overlapping read-pair observations averaged 2.36 million observations per assembly with a minimum of 74.0 thousand and maximum of 7.35 million observations. The allele-specific error test—based on the averages of allele quality scores—nearly matched the theoretical expectation as well. *Thus, both assembly-specific and allele-specific tests can be trusted to filter false variants generated by sequencer error alone.*

Unfortunately, a 99.9 % confidence interval cannot tell us how often real variants will be called significant; therefore, we have tested this empirically. For variants that ought to be real (green, Table 2), the allele-specific test is more sensitive than the assembly-specific test. However, either test is able to call 95 % of real variants significant for allelic frequencies down to 1 %. *Combining both tests together reduced the rate of calling false variants significant at a 0.5 % expected frequency (where false positives often appear should one relax heuristics) while still calling 95 % of real variants significant at a 1 % expected frequency*. Variability in the mixing process affected the observed frequencies and standard deviations of the mixed alleles in a mix-in percentage dependent manner (see Additional files 4 and 5).

### Visualization of linked variants

Computational methods for haplotyping the human genome have been well-studied [19]. For viral deep sequencing, variant phasing refers to the co-occurrence of single nucleotide variants (SNV) within the same gene segment transcript as opposed to a chromosome. Unlike the mammalian case, each read could represent the gene of a unique virus in the population, and any combination of variants may be possible. Therefore, it is of interest to know when the co-occurrence of SNVs is enriched with respect to their single nucleotide (independent) frequencies. This problem is analogous to the task of identifying associated word pairs in the field of text mining. Therefore, we have borrowed some of these association measures [20, 21] to apply to the visualization of phased viral SNVs (see section Qualitative phasing of linked variants (Fig. 1b, step 9)).

Groups of in-phase variants can indicate viral sub-populations, co-infection, or low-level contamination. For a long read assembly, it may be possible to identify phasing patterns by looking directly at the IRMA-provided assembly files; however, on a short-read assembly, complex statistical information must be used to piece together phasing information. For high-throughput NGS surveillance, where hundreds of gene segments maybe examined per run, a quick and easy-to-understand picture is required; thus, we have transformed phasing information into the well-known heat map.

**Fig. 7:**
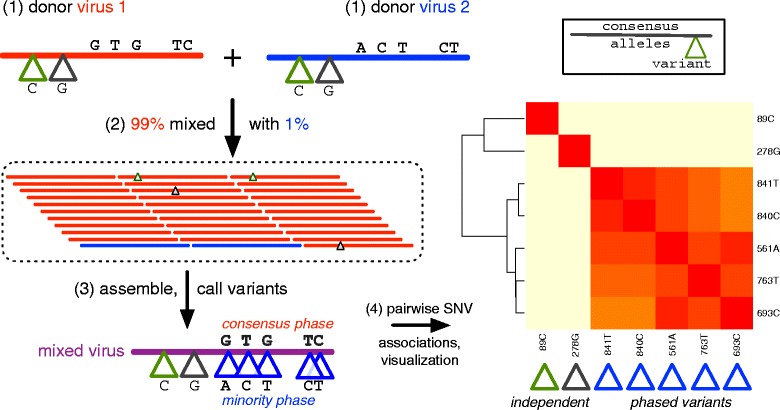
The artificial mixture, variant calling, and phasing of variants for an influenza A(H3N2) *M* gene. Donor viruses (1) are mixed (2) in a 99:1 ratio with new variants called (3) for the mixed virus and pairwise tested for phasing and visualization (4) by heat map. Consensus and minority phases are colored *red* and *blue* corresponding to the consensus alleles of each parent donor virus. Single nucleotide variants are shown with a triangle and colored according to their phase. Independent phase SNVs—without linkage to other minority variants—have *green* and *gray* triangles

IRMA clearly visualizes phased variants for the expected phases of our artificial mixture of H3N2 viruses. Figure 7 depicts the mixture and visualization process. In step 1, consensus differences and SNVs are illustrated for each donor virus’ matrix protein gene segment. After each virus is mixed (step 2), the 1 % fraction become the minority phase in the resultant assembly (step 3). Called variants are pairwise compared for linkage associations and rendered as a heat map (step 4) where the minority phase (1 % fraction) clusters in red. IRMA generates three different association measures for each assembly plus a joint frequency heat map, in case one measure performs better than another. However, in our experience the mutual dependency distance measure works quite well and is used for all figures. In addition to heat maps, IRMA automatically generates coverage/variant plots (Additional file 6*;* Fig. 1b step 9). The remaining minority phases for 7 of the 8 mixture ratios are pictured in Additional file 7. The 8^th^ mixture or 0.5 % fraction did not generate a heat map since the minority phase SNVs were not called in this replicate. This was not true of the other experimental replicates on the same gene and mixture (data not shown), suggesting variant-calling criteria can introduce a trade-off between specificity and sensitivity at lower limits of detection.

Phasing on short-read assemblies can be more challenging as gene segments get longer. This is because distant SNVs will eventually fall on different reads. Fortunately, a heat map can remain informative even when gene segments are longer. One can use the heat map to find mutually linked variants between clusters of phased variants and infer, transitively, that the clusters themselves may be linked. This concept is illustrated for a 90 to 10 % mixture of neuraminidases from the H3 experiment and is given in Additional file 8.

False variant phases can be introduced by sample contamination or barcode bleed-over. Sorting reads by gene segment and subtype can help mitigate this problem. IRMA accomplishes this task by sorting reads (Fig. 1b, step 4) using LABEL, an accurate (Fig. 3, Additional file 1) machine learning approach that rejects non-flu-like reads (Additional file 2). Moreover, by performing an all reference versus all read match step (Fig. 1b, step 3) prior to determining the best match, IRMA can utilize sub-optimal matches to identify and filter out chimeric reads, reducing assembly noise. IRMA’s read gathering phase (Fig. 1b, steps 1–5) aids variant calling by increasing coverage depth and breadth. Read mapping is optimized by Smith-Waterman (Fig. 1b, step 6) in order to increase assembly accuracy. In the face of rapidly evolving viral pathogens, these features provide high-throughput, accurate variant detection and phasing for public health surveillance programs.

## Conclusion

Our study has shown that NGS methods designed with eukaryotic and prokaryotic genomes in mind frequently do not perform as well for reference-based assembly and analysis of influenza, with implications to other segmented RNA viruses. Non-iterative approaches improve when references from the same clade are available and utilized, but for rapidly evolving organisms like influenza, it is often undesirable to rely on anecdotal reference choices as coverage may be rapidly diminished. By using iterative refinement during read gathering, sensitivity to distant genetic variants is increased. Alignment-based read-pair merging, alternative consensus generation, and the statistical evaluation of minor variants can reduce assembly error, help weed out false positive minor variants, and allow for extended linkage between variant sites.

IRMA robustly automates iteratively-refined assembly of NGS data using features designed for RNA viruses like influenza. Without the need to specify a reference beyond selecting a pre-built module, IRMA gathers reads and polishes assemblies for viral genomes using a modular, flexible strategy that improves NGS data analysis outcomes for rapidly evolving viruses, especially those with segmented genomes. IRMA features include: (1) per segment insertion, deletion, single nucleotide variant, coverage, and full allele statistics in tabular form that are easy for database ingest or human readability; (2) amended consensus files with mixed base calls (customizable) and phasing distance matrices; (3) a read count table for primary and secondary data that makes it easier to infer if there might be mixed subtypes in the sample; (4) per segment figures for read counts, coverage diagrams with highlighted SNVs, and phasing heat maps; and (5) per segment VCF files and IGV-ready assembly files via Samtools.

Because pre-built modules can be generated using existing sequence data, IRMA is readily adaptable for the analysis of both long and short-read sequence data derived from other RNA viruses, particularly those that have segmented genomes. We have previously demonstrated that IRMA works well for non-segmented RNA viruses of extreme public health consequence such as ebola [10], and have had preliminary success with the very long MERS-CoV genome (data not shown). NGS studies of other pathogens of high consequence—hantavirus, rift valley fever virus (RVFV), Crimean-Congo hemorrhagic fever (CCFV)—would likely benefit from the IRMA iterative refinement approach to virus assembly.

## Methods

### Sequencing & analysis

#### Influenza A(H3N2) mixtures

##### Sequencing and mixture protocol

RNA from two H3N2 viruses A/New Hampshire/04/2015 and A/New York/03/2015 were extracted using the QIAamp Viral RNA Mini Kit (Qiagen). The full influenza genome was amplified using the Uni/Inf primer set [22] with SuperScript III One-Step RT-PCR with Platinum Taq High Fidelity (Invitrogen). Following amplification, the amplicons generated from the two strains were quantified with Quant-iT dsDNA High Sensitivity Assay (Invitrogen) and normalized to 5 ng/ul. In triplicate, the 5 ng/ul amplified product of A/New York/03/2015 was added to the normalized stock of A/New Hampshire/04/2015 using the following percentages: 0, 0.5, 1, 2, 5, 10, 20, 25, and 50.

Indexed paired end libraries were generated from 5 μL of 0.2 ng/μL amplicon pool using Nextera XT Sample Preparation Kit (Illumina, San Diego, CA) following the manufacture’s protocol. Library reactions were purified with 0.8X AMPure XP beads (Beckman Coulter Inc.), quantified using the Quant-iT dsDNA High Sensitivity Assay (Invitrogen) and evaluated for fragment size using the QIAxcel Advanced System (Qiagen). Libraries were diluted to 2nM and pooled for sequencing. Six pmol of pooled libraries were sequenced on the Illumina MiSeq with a MiSeq v2 300 cycle kit with 5 % PhiX spike in to increase sequence diversity.

##### Calibration calculations

Given any replicate-gene-position-allele, the expected frequency was equal to the dot product of the mixture frequencies and the observed frequencies in the unmixed parent donor controls. For the control assembly, we used IRMA with both donor virus consensuses as the references (one-round each) for read-gathering and polishing assembly. Significant figures were applied based on the number of observations or, maximally, three significant figures based on the wet lab protocol. Absolute error and relative error were calculated for each aligned position and *indel* error was excluded.

#### Influenza A(H7N7) sample, (Figs. 4, 5 and 6)

##### Sequencing and random priming protocol

RNA from A/equine/Detroit/3/64 (H7N7) was extracted using QIAamp viral RNA mini kit, with modifications. First strand synthesis was completed using Superscript III First Strand System (Invitrogen, CA) with 50 ng of random hexamers according to the manufacturer’s protocol. dsDNA was generated with NEBNext Second Strand Synthesis Module (New England BioLabs, MA) according to the manufacturer’s protocol. Extraneous nucleotides were removed with a 2x Agencort RNAClean XP bead cleanup (Agencort, CA). dsDNA was sheared to 400 bp using a Covaris focused ultrasonicator (Covaris, MA). Illumina compatible libraries were constructed using the Ovation Ultralow DR multiplex system (NuGEN, CA) with approximately 1–5 ng of DNA. Library amplification was completed with 18 cycles of PCR with the following thermal cycler conditions: 72 °C for 2 min, 18 cycles of (94 °C for 30 s, 60 °C for 30 s, and 72 °C for 1 min), 72 °C for 5 min, and hold at 10 °C. The NuGEN supplied single index barcodes were used according to the manufacturer’s guidelines. Amplified libraries were purified using a 0.8x Agencourt RNA bead clean up and eluted in 33 μL of 1x low TE. Libraries were quantified using the Qubit dsDNA high sensitivity assay (Life Technologies, CA). Libraries were diluted to 2nM in preparation for pooling and denaturation for running on the MiSeq (Illumina, CA).

Pooled and NaOH denatured libraries were diluted to 10 pM and sequenced on an Illumina MiSeq with 2 × 150 bp paired end reads using the MiSeq v2 300 cycle kit or 2 × 250 bp paired end reads with the MiSeq v2 500 cycle kit (Illumina). Five percent PhiX (Illumina, CA) was included in each run.

##### Analysis of iterative assembly (Figs. 4, 5 and 6)

The known HA and NA consensuses or “baselines” were identical to public sequences KF435057 and KF435059 respectively. The “consensus” reference templates corresponded to the N7 and H7 alignment majority consensuses as mentioned in (section Influenza alignment dataset), which is the standard IRMA reference set. GenBank accessions for the “same clade” reference, “distant clade” reference, and “divergent branch” reference were CY130134, KF258958, CY034190 for hemagglutinin and CY130136, CY094767, and KF259666 for neuraminidase respectively.

IRMA used defaults except for the maximum read gathering iteration and with the specified reference sets. Maximum iterations for the final assembly stage (Fig. 1b step 6, using SSW) were set to the default of 5. Bowtie2 was run using a very-sensitive, local assembly with the resultant statistics and read-pair merging calculated using the same scripts as IRMA to normalize read depth comparisons.

### Datasets

#### Influenza alignment dataset

To create the influenza A virus reference sequence alignments, publically available genetic data for each subtype and internal gene was downloaded from GenBank and aligned. Neighbor joining phylogenetic trees estimated with an HKY-85 nucleotide model were produced in PAUP v4.0a146 [23] to identify and remove singleton outliers. Internal gene segments were initially downloaded according to *HA* type. After removing singletons, these datasets were combined and realigned.

Influenza B sequences were also downloaded from GenBank. Downstream processing for influenza A and B included removal of duplicate sequences, sequences with greater than five ambiguous nucleotides, sequences causing frame-shifts, and short sequences (less than 60 % alignment length), were also removed using custom scripts. Final alignments were created using MAFFT v7 [24] and edited in JalView v2.8 [25]*.* A total of 165,470 sequences were obtained for the 39 gene segment groups (8 B segments, 6 internal A segments, 16 *HA* subtypes, and 9 *NA* subtypes). Plurality consensus sequences were taken for each alignment for IRMA’s default reference set for the influenza module. Profile HMMs were also generated against these alignments using SAM [26] v3.5, for use in LABEL (see Sort step (Fig. 1b, step 4)) or for SAM rough alignment (see Align step (Fig. 1b, step 5); also Fig. 1b step 5).

#### Influenza A(H3N2) genetic diversity, (Fig. 2)

We down-sampled the H3 alignment to only include sequences from 2012 and performed pairwise distance matrix calculations using a custom script. We annotated sequences according to human, swine, or other host and averaged the pairwise distances between and within each host group. For the full lower triangle of the pairwise distance matrix, we created a density plot in R for visualization in Tableau. The phylogenetic tree (maximum-likelihood, GTR + GAMMA, 10,000 local support bootstraps) on the same dataset was calculated using FastTree v2.1.8 [27] with figure generation via FigTree v1.4.2 program. The same tools and settings were also applied to the trees in Fig. 6.

#### Random subsequence datasets (Fig. 3 & Additional file 1)

Three subsequence datasets were created using random sampling with replacement and from randomly chosen regions (subsequence) within each sequence (custom scripts, available upon request). The first dataset was used to assist LABEL training and set the inappropriate data filters. We will talk of “positives” as our influenza data. For negatives, we used over 23 k unique viral sequences from Isavirus, Orbivirus, Respirovirus, Rotavirus, and Thogotavirus genus groups, obtained from GenBank. Using custom scripts, 6000 subsequences of length 50 from each genus group were sampled and de-duplicated. For the positive dataset, we sampled 6000 subsequences of length 150 (typical for short reads) from each influenza gene segment and subtype in the alignment dataset—removing duplicate sequences. We obtained 205,837 positive subsequences (influenza, length 150) and 27,245 negatives (non-influenza, length 50). Shorter length subsequences are harder to classify, hence the conservative use of length 50 for the negatives.

The second dataset was used for Fig. 3. Using a complete re-sampling and following the same procedure as the first dataset but with longer negatives, we obtained a positive set of 205,873 influenza subsequences, and a negative set of 28,164 non-influenza subsequences—both of length 150. We characterized the number of nucleotide differences between each subsequence (query set) and its alignment consensus (target set) and then tried to match the query data to consensus using various programs: BLAT [28] v35x1 with one mismatch allowed, minimum identity 80, tile size 10; YARA [29, 30] v0.9.3 with the maximum permitted error rate/edit distance 10; Bowtie [31, 32] v2.2.4 using local & end-to-end very-sensitive searches; and MOSAIK [33] v2.2.3 with neural network files version 2.1.78 & default settings. For MOSAIK, *fasta* data was converted to *fastq* and given maximal quality scores for each base (since they are known). We also used the LABEL [11] v0.4.5 (module “irma-FLU”) to classify subsequences into influenza A and B gene segments and subtypes. LABEL rejects queries if they do not pass an inclusion criterion (see Additional file 2). None of the programs falsely returned hits for the negative control (data not shown), therefore, only sensitivity to influenza was shown in Fig. 3. For Fig. 3 panel C, we varied BLAT’s minimum identity parameter on the same dataset used for panels A and B. We chose BLAT for Fig. 3c because it was the most sensitive non-machine-learning approach.

The third dataset was uniquely sampled from the 39 influenza alignment consensus sequences, obtaining 500 for each consensus or 19,500 subsequences. Each subsequence was randomly mutated at 9 fixed levels (1, 5, 10, 15, 20, 25, 30, 35, & 40 substitutions) and combined for a total of 175,500 influenza subsequences. For LABEL, we tried a second set of modules: *irma-FLU-HA*, *irma-FLU-NA*, and *irma-FLU-OG* as a composite classifier (called “trio”, see Additional file 2) for hemagglutinin subtypes, neuraminidase subtypes, and all other genes respectively. Results for this dataset were shown in Additional file 1.

### Iterative refinement meta-assembler algorithm (Fig. 1b)

Key IRMA features include: (1) per segment insertion, deletion, single nucleotide variant, coverage, and full allele statistics; (2) amended consensus files with mixed base calls (customizable) and phasing distance matrices; (3) a read count table for primary and secondary data; (4) per segment figures for read counts, coverage diagrams with highlighted SNVs, and phasing heat maps (see Additional file 6); and (5) per segment VCF files and IGV-ready [34] assembly files via Samtools [35].

The IRMA pipeline, may use either paired-end read files (Illumine platforms) or a single unpaired read file (PacBio, Ion Torrent, *et cetera*). Parameterization of the pipeline relies on named, customizable configuration files to tweak performance, assembly, or variant calling. We discuss default parameters (in parentheses), options, and how the algorithm proceeds. An outline of the IRMA influenza module is given in Fig. 1b.

#### Iterative read gathering (Fig. 1b, steps 1 to 5)

Reads may be filtered based on sequence length (minimum 150 nucleotides) or read median quality (minimum 30). It is also possible to use an average quality calculation instead of a median one, but evidence favors the use of a median to overcome outliers [5]. In the read gathering phase, IRMA performs an all-versus-all match, sorts out the best match, rough aligns and generates an intermediate consensus. We refer to these steps as the match, sort, and align steps. Some programs may combine these steps together, but it is sometimes useful to pull them apart.

##### Match step (Fig. 1b, step 3)

After quality control filtering, reads are de-duplicated into *read patterns* (for efficiency). BLAT is used to match all read patterns against all references on both strands. Chimeric reads—read patterns matching both strands on the same gene—may be discarded. Multiple references per gene are allowed if of the same length.

##### Sort step (Fig. 1b, step 4)

Read patterns matched to the reference set are sorted into their best gene match. We used LABEL as a default to classify reads into their *HA* subtype, *NA* subtype, or other gene (*PB2*, *PB1*, etc.). LABEL’s inappropriate data filter can separate non-*HA*-like, non-*NA*-like, and non-other-gene-like read patterns (Additional file 2). BLAT scoring (match minus mismatch) can also be used as a faster option to sort reads (combining the match & sort steps, which is typical of many assembly programs).

It is worth noting that for two or more samples being multiplexed, barcode bleed-over can introduce low-level, linked variants within regions of high similarity. Such reads contain real signal but mislead variant calling efforts. Highly accurately sorting of reads into best match gene bins helps eliminate such issues—where sorting is possible at the short read level. The current IRMA/LABEL influenza modules sort at the type and subtype levels, but one could build more specific classifiers.

##### Align step (Fig. 1b, step 5)

After sorting into the best match gene, read patterns are aligned to the gene-specific reference or profile HMM. We use SAM as default to align read patterns against profile HMMs, BLAT optionally. At this stage statistics are calculated, merged and a new consensus sequence generated (plurality).

##### Reference elongation, deletion editing, and alternative consensus generation

The consensus from the first iteration may be used to gather reads in the next iteration, although typically only two rounds (five is our new default, two was used in this study except where indicated) are necessary. Optionally, IRMA can elongate the references (used in the match step) by counting the bases past the 5′ and 3′ ends and using the aligned data as an anchor. The elongation stops when the consensus count (plurality) at either end is less than one fourth (minimally ten counts) the count of the last or first non-elongated consensus base respectively. Reference elongation is useful for UTR discovery, but is dangerous if ends are very conserved.

In addition to reference elongation, deletion editing occurs if the consensus allele is in a deleted state. One can optionally delete by ambiguation—replacing the reference base with an ‘N’ nucleotide—to retain the same reference size. This option is helpful when using BLAT in the align step instead of SAM.

Finally, an alternative or secondary reference can be optionally generated alongside the regular alignment consensus. In the alternative reference, the second most frequent allele is used at each site, instead of consensus, so long as it is sufficiently frequent (count ≥ 20, frequency ≥ 2 %). If no sites pass threshold, a secondary reference is not created even if these thresholds are set.

##### Finishing read gathering

IRMA iterates on gathering read patterns, sorting by gene segment, and aligns them to create a more accurate reference, stopping when the maximum iteration is reached (five rounds), or when no more reads can be matched to the primary gene segments. For influenza, the presence of influenza B or A, and various *HA* and *NA* subtypes allows for multiple gene segments to be present. If more than one type or subtype are present for the same gene segment, they are categorized as primary or secondary based on whichever is a plurality. Secondary or alternate read patterns, sorted to gene segments of a different type or subtype, are tabulated and stored alongside the main results but do not undergo final assembly. Consensus sequences for the primary data are passed to iterative final assembly.

#### Iterative final assembly (Fig. 1b, step 6)

Starting with the consensus sequence from the read gathering phase, we use the SSW implementation [36] of the Smith-Waterman [37] algorithm to finalize assembly (this implementation is faster than the original, but is also run in parallel by IRMA to scale with the task at hand). Reads are re-created from read patterns and assembled using iterative refinement of the reference and SSW scores. We do not trim reads based on quality scores; rather, we trust Smith-Waterman to hard cut reads when they no longer align to either the primary or alternative consensus (if generated).

At each assembled site, if a deletion threshold (minimum 50 %) or insertion threshold (minimum 15 %) is reached, a reference edit is tried in the next iteration and the score compared with the previous round. If the total assembly score is reduced, matches the previous iteration (converges), or reaches a maximum iteration (5 rounds), the iterations stop and the highest scoring round is used. Please note that IRMA’s SSW iteration & optimization procedure refers to the running of *full* SSW assemblies in rounds separated by reference editing, and should not be confused with the dynamic programming algorithm used to optimize read alignments within an individual Smith-Waterman run.

Just as a secondary reference can be generated in the read gathering phase, an alternative reference may also be created in the final assembly phase (using the same thresholds as before). Reads are matched to both references and the read alignment which maximizes the Smith-Waterman score is kept.

At the end of the Smith-Waterman iterations, if read-pairs were used, each gene segment undergoes a reference based read-pair overlap merging which we will discuss in the next section. The merged final assembly is used to create final consensus, variants, and any variant phasing/linkage calculations. All allelic tabulations, including insertion and deletion tables, coverage tables, and VCF files are also generated from this final assembly and its consensus. Amended consensus sequences—which give ambiguous nucleotide codes for mixtures where the called minority allele has achieved a frequency threshold (minimum 25 %)—are output as additional consensus views of the assembly.

#### Reference based read-pair merging and correction (Fig. 1b, step 7)

For Illumina paired-end reads, merging read-pairs prior to assembly via probabilistic modeling and alignable overlap has been shown to be beneficial [38, 39]. Since viral genomes are very small, we prefer to do a reference based read-pair merge, *post* assembly instead of prior to or during assembly. Each read-pair is independently aligned to the final reference consensus and the pairs merged with or without the presence of an overlap (which benefits phasing on short reads). Overlapping regions can be corrected parsimoniously using both the reference alleles and obvious differences in quality (see Additional file 9). K-mer based methods [40] are also popular for error correction, yet because of high viral mutation rate, we did not want to unnecessarily permanently remove viral diversity based on frequency.

Since both read-pairs are from the same molecule, any time overlapping bases disagree they represent a sequencer error with certitude. The read-pair overlap disagreement rate can be used in variant calling as an assembly-specific estimate of sequencer error.

#### Variant calling (Fig. 1b, step 8)

Minor variant alleles were called when they rejected the null hypotheses for all confidence interval tests as well as exceeded heuristic thresholds. For confidence intervals, we used a 99.9 % one-sided second order corrected binomial confidence interval [41] around an allele-specific error estimate (mean allele quality score) and an assembly-specific error estimate (when available, read-pair overlap disagreement rate, for each assembly). For the quality based error estimate, as in [7], we used coverage for the number of trials and an average quality score, but unlike Wright et al. we averaged quality scores over the minor allele in question rather than the site, since each allele can have a different error profile. If the observed allelic frequency exceeded both of the interval upper bounds, we counted them as significant with respect to sequencer error. The choice of a corrected interval was to avoid conservative coverage probabilities in order to limit false negatives.

For heuristics, variants were required to have a minimum frequency (0.8 %), minimum average quality (24 quality score), minimum allele count (default 2), minimum site depth (default 100), and minimum confidence (80 %). Confidence is the ratio of the estimated frequency to the observed allelic frequency. The estimated frequency was the observed frequency less the allele-specific error estimate based on the average allele quality score. If the allele-specific error estimate was greater than the observed frequency then the estimated frequency was zero. As the error rate goes to 0, confidence goes to 100 % and the relationship between observed and estimated frequencies become linear. An automatic heuristic adjustment of the minimum variant frequency (default is *on*) sets the threshold to the maximum of 0.8 % or the most frequent minor allele with a zero confidence.

#### Qualitative phasing of linked variants (Fig. 1b, step 9)

For each called variant, pairwise joint frequencies and individual frequencies were calculated. We used a slightly modified Jaccard distance (formula A.15), a mutual dependency distance (formula A.16), an experimental enrichment measure (formula A.17) inspired by di-nucleotide enrichment [42], and a joint frequency distance (formula A.18). Formulas and supplementary methods for phasing calculations and allele statistics are listed in Additional file 10. If the assembly used read-pairs, merged pairs were considered to be linked regardless of overlap, allowing for phasing at greater distances than for unpaired short read assembly (this is not necessary for long read technologies).

We provided qualitative linkage/phasing analysis by way of heat maps. Heat maps were generated in R from pairwise distance matrices over all called SNVs and for each distance measure. Distances were in the unit interval with total co-occurrence at zero and the greatest lack of linkage at one. Linked minor variant form clusters in the heat map while unlinked minor variants only appear on the diagonal. Transitive relationships (A co-occurs with B and B with C, therefore A with C) may be inferred but not with certitude unless phasing is at maximum (Additional file 8). Given short read assembly, sensitivity to phased variants will be more difficult as the distance between them increases. Inference of linkage using transitive properties and heat maps may provide additional insights suitable for testing.

#### Insertion and deletion variants

Insertion variants were filtered using the same heuristics as SNVs for quality score, variant count, confidence, and overall read depth support. However, for insertions, the average quality score was the average of mean quality for each insert over its length. The total depth was calculated only where reads contained flanking nucleotides. Insert positions were relative to the upstream base. The same statistical confidence intervals (Additional file 10) were used but with error estimates for paired-end insertion error rate (Additional file 9) and the average insert quality score. Minimum insertion variant frequency thresholds (default 0.5 %) are customizable and distinct from deletion and single nucleotide variants frequency thresholds.

Deletion variants were also called relative to the upstream base. Flanking non-deleted alleles were required for each read and deletions of different length were treated as different deletion variants. (For completeness, deletion allele statistics, irrespective of length, were recorded at each position in the “all alleles” tables along with each canonical allele.) Deletions, by their nature, could not be called using quality score information but deletion observation count, total depth, and a minimum deletion variant (default 0.5 %) frequency could be used. Likewise, only a paired-end deletion error rate could be used for confidence interval tests.

## Availability of supporting software and data

IRMA may be downloaded from wonder.cdc.gov/amd/flu/irma along with the read data used in the H3 mixture study. We also document our software—including usage, parameters, grid execution, and module creation—on the home page. IRMA is written in BASH and Perl and requires a Linux or Mac OS X system. For single computer multi-core performance characteristics, see Additional file 11. IRMA may also be run on Open/Sun/Univa Grid Engine for cluster acceleration. Some of IRMA’s local parallelization was provided by GNU Parallel [43] v20150222. Packaged modules, at the time of writing, include influenza and ebola virus.

## Additional files

Additional file 1: Sensitivity to artificial influenza diversity. Line plots show normalized frequencies at each fixed mutation level (1, 5, 10, 15, 20, 25, 30, 35, 40) and method and are characterized as sensitive to flu (matched to correct consensus), rejected (not matching any consensus), or misclassified (matching incorrect consensus). Tabular summaries represent the proportion for each method across all 175,500 subsequences irrespective of mutation level. The dashed vertical lines represent a general limit of detection for the given approaches. (PDF 141 kb) Additional file 2: Inappropriate data filtering for the “irma-FLU” LABEL module as well as the trio of modules “irma-FLU-HA”, “irma-FLU-NA”, and “irma-FLU-OG”. These modules reject reads as unrecognizable if they fail to pass filtering criteria. Density and strip charts are shown for positive and negative data for each module along with the filtering threshold. (PDF 1144 kb) Additional file 3: Average absolute error, standard deviation and sum of absolute error (bracketed) are shown for each group & parameterization in (A). Observations are the total number of assembled nucleotides for all genes and mixtures in triplicate. Allele type is per mixture-replicate-gene-position showing consensus (99.994 % majority, 0.006 % plurality) allele error and minority allele (non-consensus) error. For the extra parameters, “BLAT” refers to the use of BLAT for sorting instead of LABEL, “flat” refers to uniform Smith-Waterman weights, “no merge” means read-pairs did not undergo overlap merging, and “alternative ref” means a second reference was dynamically generated based on minor variation. Average relative error is shown in (B). (XLSX 33 kb) Additional file 4: Mean average frequency and mean standard deviations of alleles appearing in all three replicates. Default IRMA assembly on calibration data. Consensus alleles were a plurality. Major mixtures had one parent donor allele with ≥98 % and the other ≤1 %. Non-mixtures were unmixed or were alleles where both donors had ≥98 % or both had ≤1 % frequency. One unmixed parent assembly was removed from mixture level = 0 because of a problem with one of the replicates. (XLSX 30 kb) Additional file 5: Variability due to the artificial mixture protocol. The observed mixture frequency versus the mix-in percentage is shown for sites and alleles where the unmixed donor viruses had had frequencies ≥98 % or ≤1 %. (PDF 6010 kb) Additional file 6: IRMA figure output. The read percentage chart, coverage/variant diagram, and phasing heat map produced by IRMA are displayed and explained. (PDF 166 kb) Additional file 7: Phasing information for artificially mixed H3N2 viruses. For each mixture of replicate 2, heat maps show the matrix protein’s minority phases. The minimum variant calling frequency was relaxed to 0.25 % for these mixtures. (PDF 569 kb) Additional file 8: Inferring phased clusters via the transitive property. Neuraminidase phased variants for the 9:1 mixture of replicate 2 are shown. (PDF 141 kb) Additional file 9: Read-pair merging rules for correcting paired-end reads. (XLSX 10 kb) Additional file 10: Supplementary methods and formulas used for the variant phasing. (PDF 113 kb) Additional file 11: IRMA and Bowtie2 assembly benchmarks for various parameterizations on a locally executed Intel(R) Xeon(R) CPU, X5660 @ 2.8Ghz with 6-cores or 12 threads and 111 Gb RAM. (XLSX 38 kb)

## Abbreviations

CDC: Centers for disease control and prevention
DST: Deep sequencing technology
HA: Influenza hemagglutinin gene segment
HKY-85: Hasegawa, Kishino and Yano 1985 model of evolution
*indel*: Insertion or deletion
IRMA: Iterative refinement meta-assembler
NA: Influenza neuraminidase gene segment
NGS: Next-generation sequencing/sequencer
pHMM: Profile hidden Markov model
SNV: Single nucleotide variant
UTR: Untranslated region
VCF: Variant call format file
