# Erratum to: Viral deep sequencing needs an adaptive approach: IRMA, the iterative refinement meta-assembler

**DOI:** 10.1186/s12864-016-3138-8

**Published:** 2016-10-13

**Authors:** Samuel S. Shepard, Sarah Meno, Justin Bahl, Malania M. Wilson, John Barnes, Elizabeth Neuhaus

**Affiliations:** 1 Influenza Division, Centers for Disease Control and Prevention, 1600 Clifton Road, Atlanta, GA 30329 USA; 2 Center for Infectious Diseases, The University of Texas School of Public Health, Houston, TX USA; 3 Battelle Memorial Research Institute, 1600 Clifton Road, Atlanta, GA 30329 USA

## Erratum

*n.b. The errors and associated corrections described in this document concerning the original manuscript were accountable to the production department handling this manuscript, and thus are no fault of the authors of this paper.*

In the original publication of this article [1], the yellow and green shading for Table 2 was removed, which meant that any references to the specific shading did not make sense. The original version of the table can be found below:

**Table 2:**
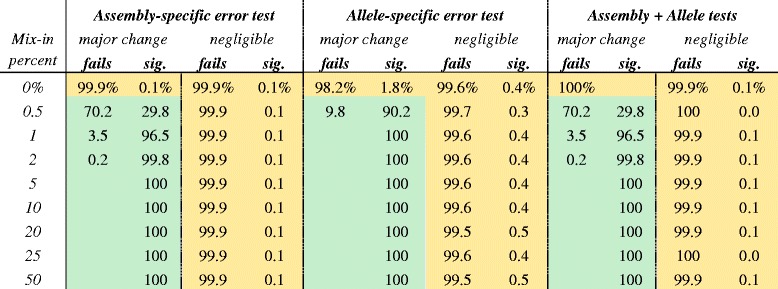
Significance testing of variant alleles on H3 influenza mixtures

Negligible allele mixtures meant both unmixed parent viruses had frequencies ≥ 98 % or ≤ 1 % while major allele mixtures were defined as one pre-mixture donor virus having ≥ 98 % frequency and the other ≤ 1 % frequency. Variant negatives (negligible mixtures or 0 % mix-in) are highlighted in yellow while variant positives are in green. Cell data were omitted when counts were zero. The null hypothesis was that variants were produced by sequencer error. All tests were with respect to second-order corrected, one-sided 99.9 % binomial confidence intervals. The percentages of minor variant alleles not distinguishable from sequencer error is marked “fails” for failing to reject the null hypothesis. The percentage of variants rejecting the null hypothesis is marked “sig.” for significant and are candidates for calling single nucleotide variants
