# Supplementary figures and images for: Viral deep sequencing needs an adaptive approach: IRMA, the iterative refinement meta-assembler
Source: BMC Genomics. 2016 Sep 5;17(1):708. doi: 10.1186/s12864-016-3030-6 (PMC5011931; doi:10.1186/s12864-016-3030-6)

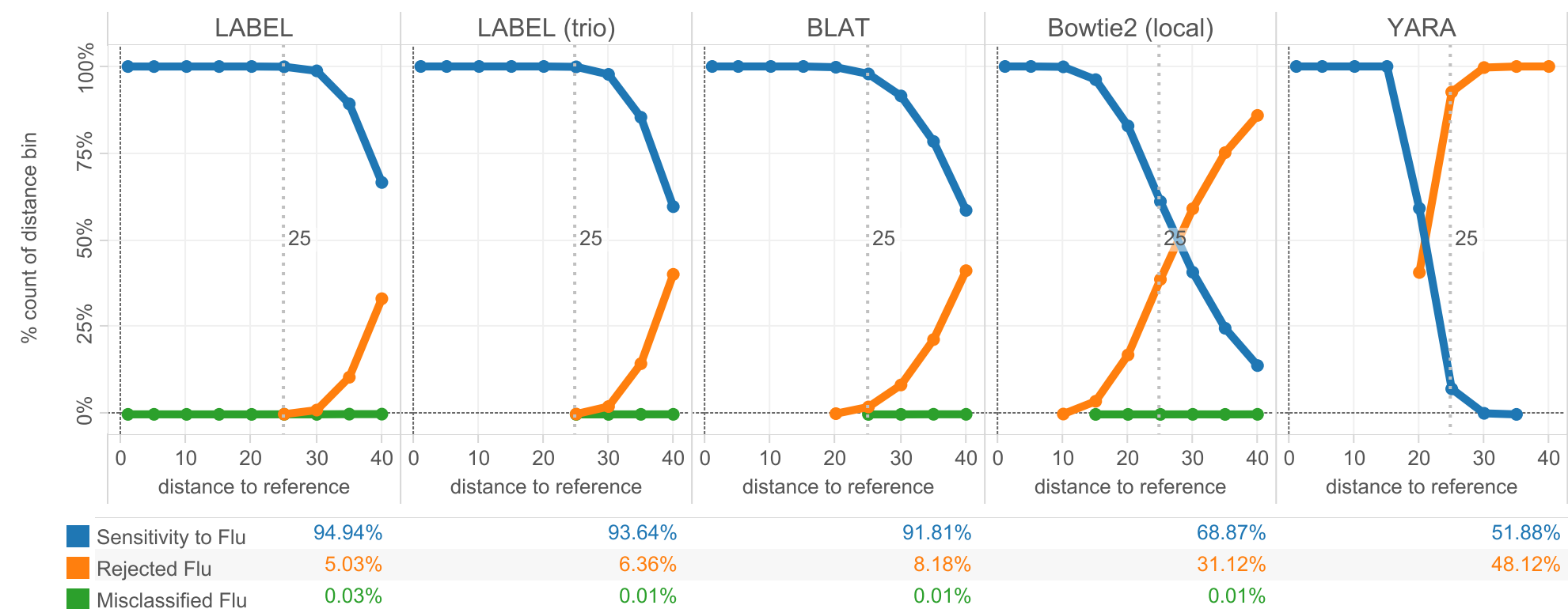

Supplement: Additional file 1: — Sensitivity to artificial influenza diversity. Line plots show normalized frequencies at each fixed mutation level (1, 5, 10, 15, 20, 25, 30, 35, 40) and method and are characterized as sensitive to flu (matched to correct consensus), rejected (not matching any consensus), or misclassified (matching incorrect consensus). Tabular summaries represent the proportion for each method across all 175,500 subsequences irrespective of mutation level. The dashed vertical lines represent a general limit of detection for the given approaches. (PDF 141 kb) [file 12864_2016_3030_MOESM1_ESM.pdf]

Variability of mixtures, alleles with major change (both parent donors  $\geq 98\%$  or  $\leq 1\%$ )

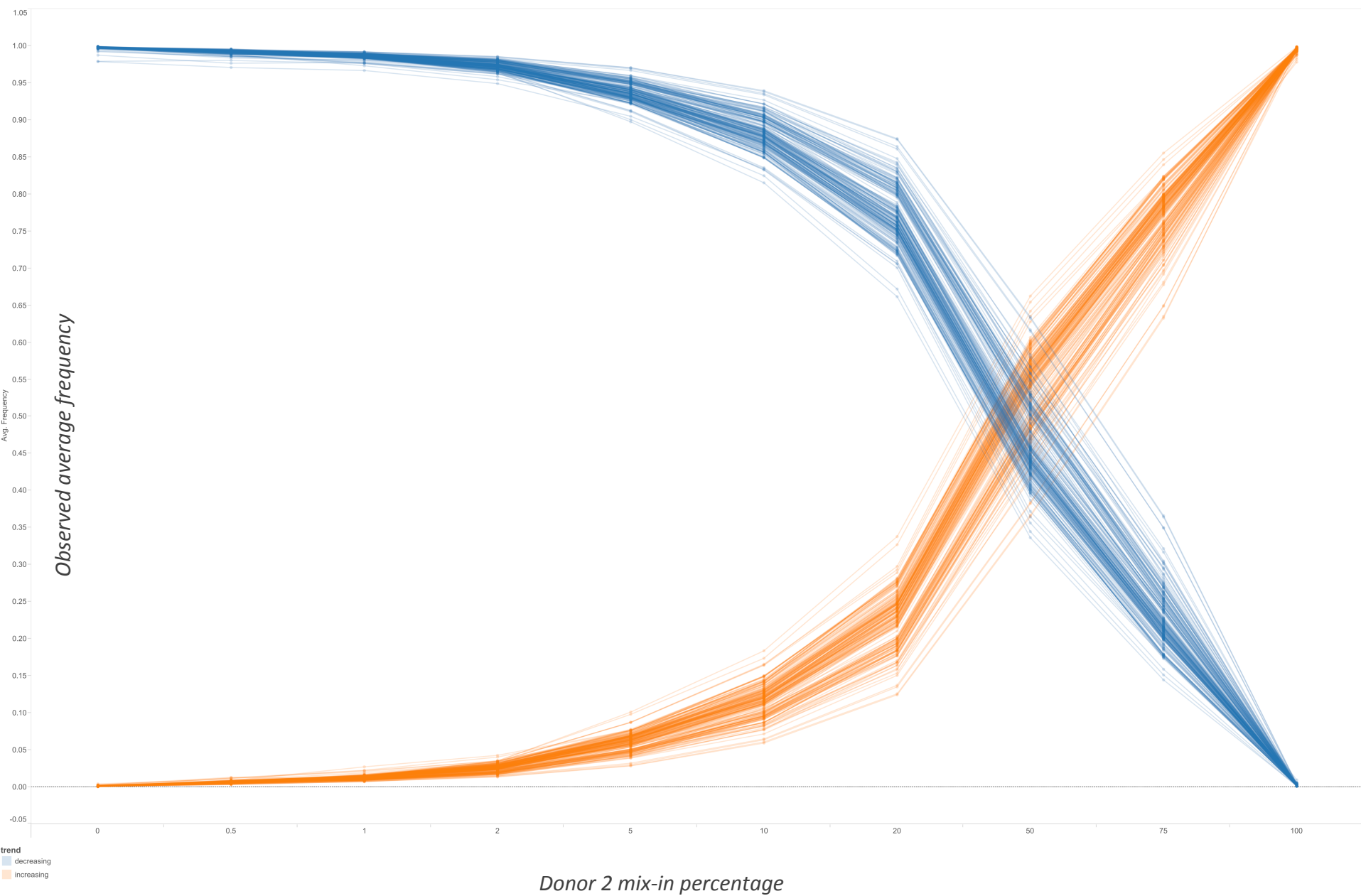

Supplement: Additional file 5: — Variability due to the artificial mixture protocol. The observed mixture frequency versus the mix-in percentage is shown for sites and alleles where the unmixed donor viruses had had frequencies ≥98 % or ≤1 %. (PDF 6010 kb) [file 12864_2016_3030_MOESM5_ESM.pdf]
