# Supplementary material for: Viral deep sequencing needs an adaptive approach: IRMA, the iterative refinement meta-assembler
Source: BMC Genomics. 2016 Sep 5;17(1):708. doi: 10.1186/s12864-016-3030-6 (PMC5011931; doi:10.1186/s12864-016-3030-6)

Inappropriate data filtering of **LABEL modules** for IRMA

ADDITIONAL FILE 2

LABEL module
irma-FLU

Classifies influenza A and B gene segments as well as 16 HA subtypes and 9 NA subtypes.

Density and jitter-plots show separation of full influenza gene sequences and subsequences versus non-flu subsequence control. Threshold used to separate non-flu-like reads.

Not used by default.

See Figure 3 and Supplementary Figure S1

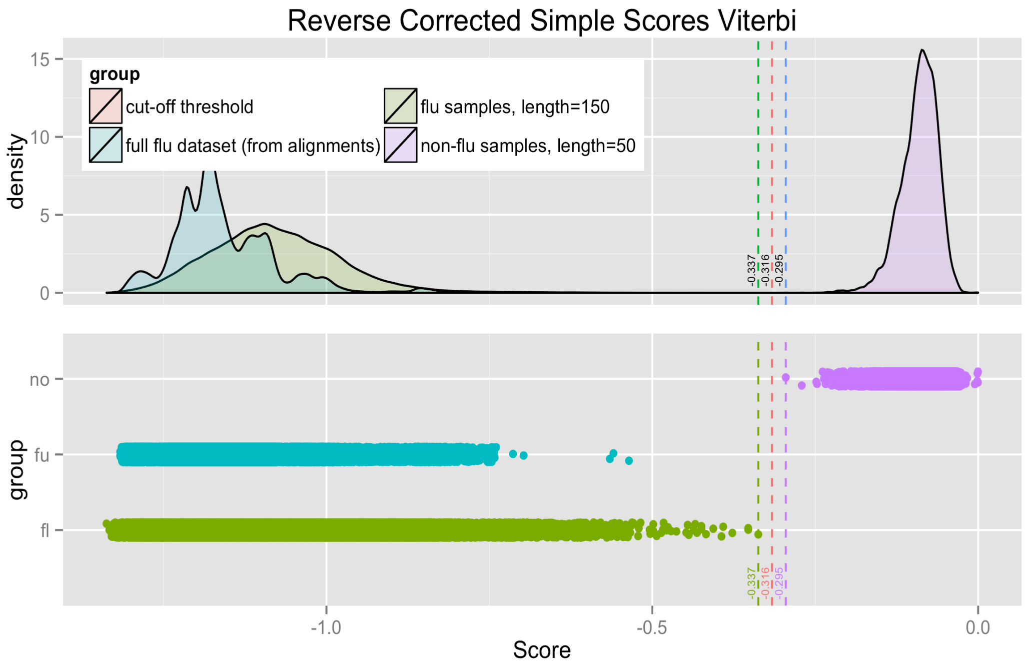

LABEL module
irma-FLU-HA

Classifies influenza A HA subtypes (1-16) and influenza B HA, given an HA.

Density and jitter-plots show separation of full influenza HA sequences and subsequences versus non-HA subsequence control. Threshold used to separate non-HA-like reads.

See Figure 3 (LABEL trio).

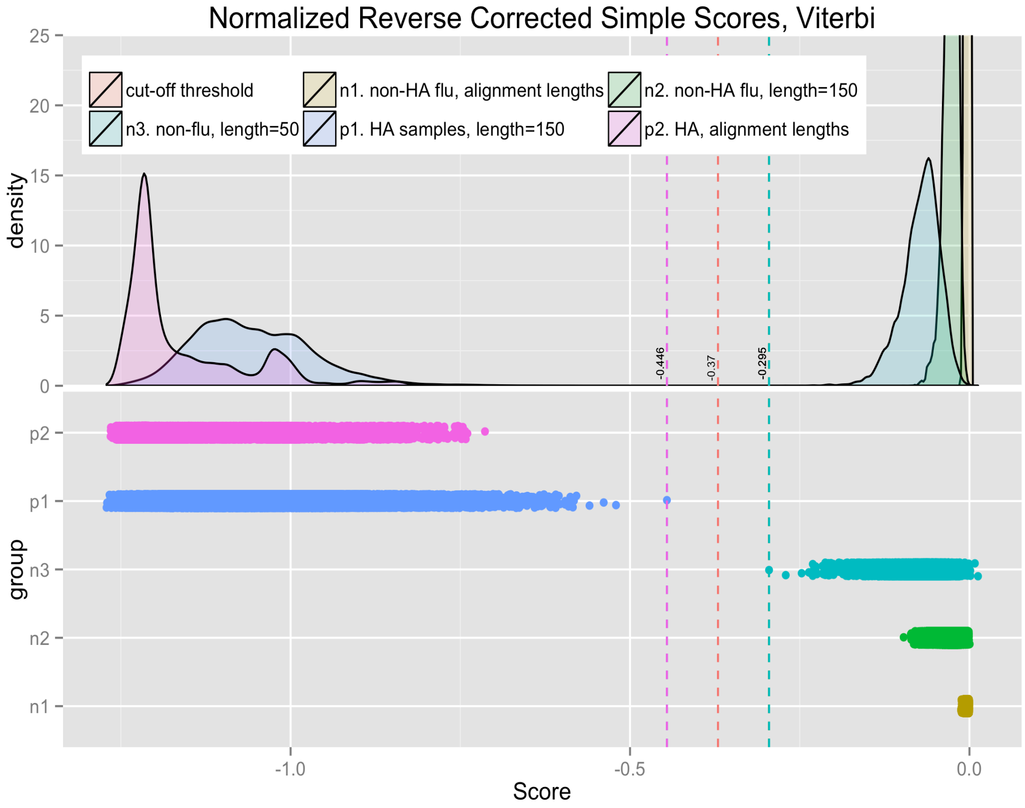

LABEL module

irma-FLU-NA

Classifies influenza A NA subtypes (1-9) and influenza B NA, given an NA.

Density and jitter-plots show separation of full influenza NA sequences and subsequences versus non-NA subsequence control. Threshold used to separate non-NA-like reads.

See Figure 3 (LABEL trio).

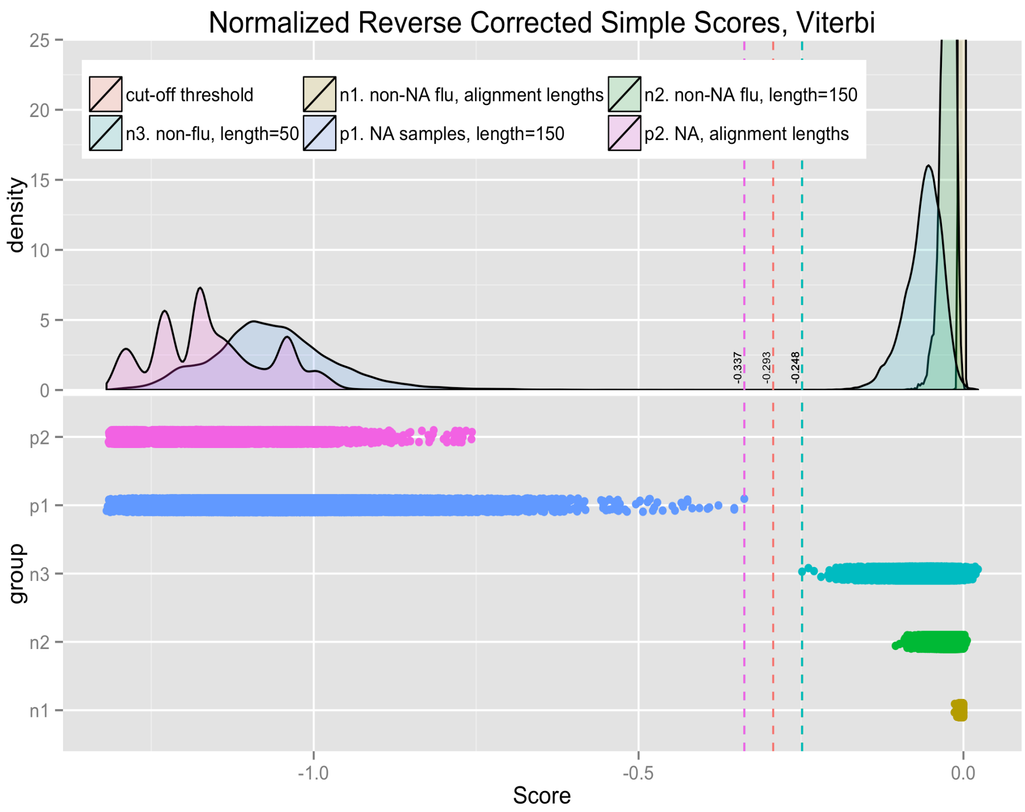

LABEL module
irma-FLU-OG

Classifies internal (PB2, PB1, PA, NP, MP, NS) gene segments for influenza A and B. Termed “other gene” or OG for short.

Density and jitter-plots show separation of full influenza OG sequences and subsequences versus non-OG (HA/NA) subsequence control. Threshold used to filter non-internal-gene-like reads.

See Figure 3 (LABEL trio).

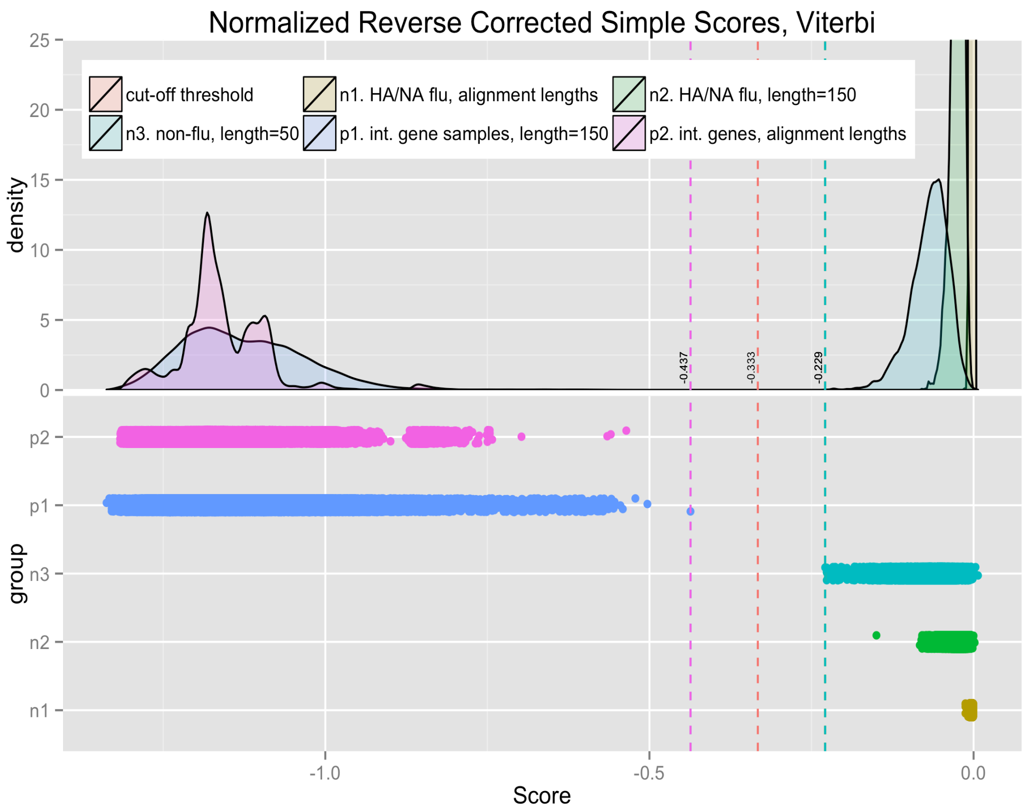

Supplement: Additional file 2: — Inappropriate data filtering for the “irma-FLU” LABEL module as well as the trio of modules “irma-FLU-HA”, “irma-FLU-NA”, and “irma-FLU-OG”. These modules reject reads as unrecognizable if they fail to pass filtering criteria. Density and strip charts are shown for positive and negative data for each module along with the filtering threshold. (PDF 1144 kb) [file 12864_2016_3030_MOESM2_ESM.pdf]
