# Supplementary material for: Viral deep sequencing needs an adaptive approach: IRMA, the iterative refinement meta-assembler
Source: BMC Genomics. 2016 Sep 5;17(1):708. doi: 10.1186/s12864-016-3030-6 (PMC5011931; doi:10.1186/s12864-016-3030-6)

Description of figures output by IRMA

ADDITIONAL FILE 6

Read percentages

1) Assembled reads versus QC filtered and additionally filtered reads
2) Percentages of read patterns passing QC versus unusable and chimeric reads
3) Percentages of merged-pair reads for each assembled gene segment

1. Percentages of total reads (R1 + R2)

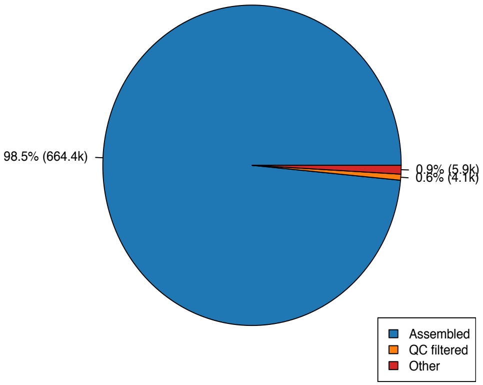

2. Percentages of all read patterns passing QC

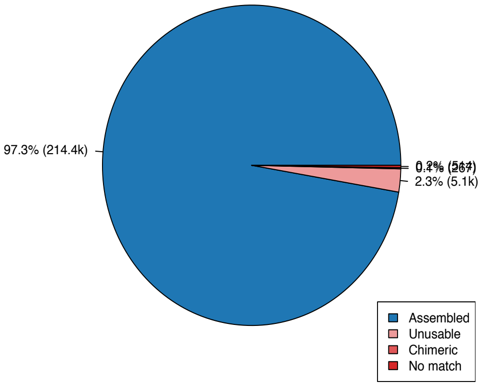

3. Percentages of assembled, merged−pair reads

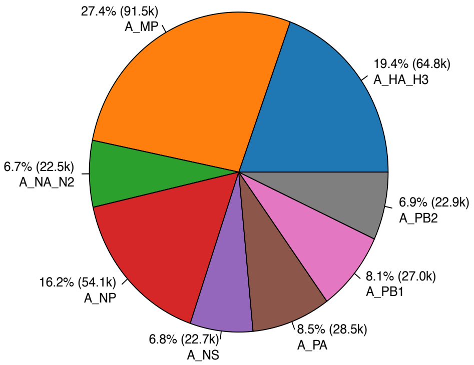

READ PROPORTIONS.

1. Percentages of total read counts (R1 & R2)
   – ASSEMBLED: influenza reads in final assemblies.
   – QC FILTERED: didn't pass length/median quality thresholds.
   – OTHER: non−flu and contaminant/poor flu signal.

2. Percentages of all read patterns passing QC process
   – Patterns are clustered or non−redundant reads.
   – ASSEMBLED: excellent influenza read patterns.
   – UNUSABLE: poor or contaminant flu patterns.
   – CHIMERIC: flu patterns matching both strands.
   – NO MATCH: non−flu read patterns.

3. Percentages of assembled, merged−pair read counts
   – Shows the proportion of gene segments to the genome.
   – Paired−end reads have been merged into a single count unless not applicable: single−end reads have been used.

Coverage diagram

(A) Read depth per site [y-axis] is depicted for the length of the gene segment [x-axis]. Called single nucleotide variants [SNV] are colored by minor allele.

(B) Observed normalized frequency is given for each SNV ordered by position and colored by minor allele. The paired-end read overlap disagreement rate is given in black. The consensus allele is given on the x-axis with the minor allele.

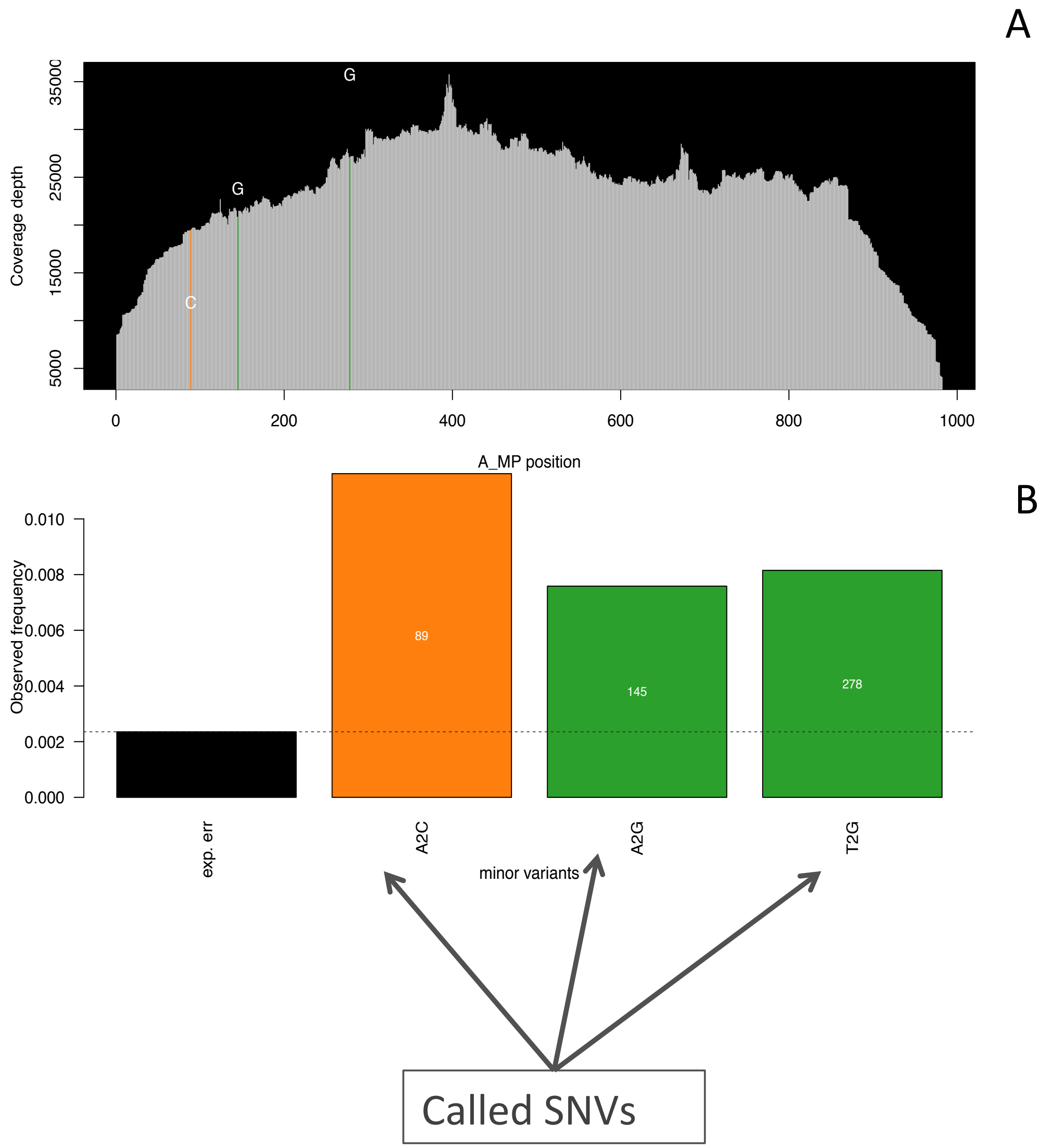

Phasing heat map

Every called single nucleotide variant [SNV] is pairwise compared to each other SNV. If the SNVs are linked, they color in **dark red** while unlinked SNVs are colored in bright **yellow**. Called SNVs are compared using four distance measures: Jaccard distance, mutual association distance, scaled and transformed joint frequency, and an experimental enrichment distance. Clustered SNVs are considered to be in phase or linked to the same viral sub-population segment, qualitatively speaking.

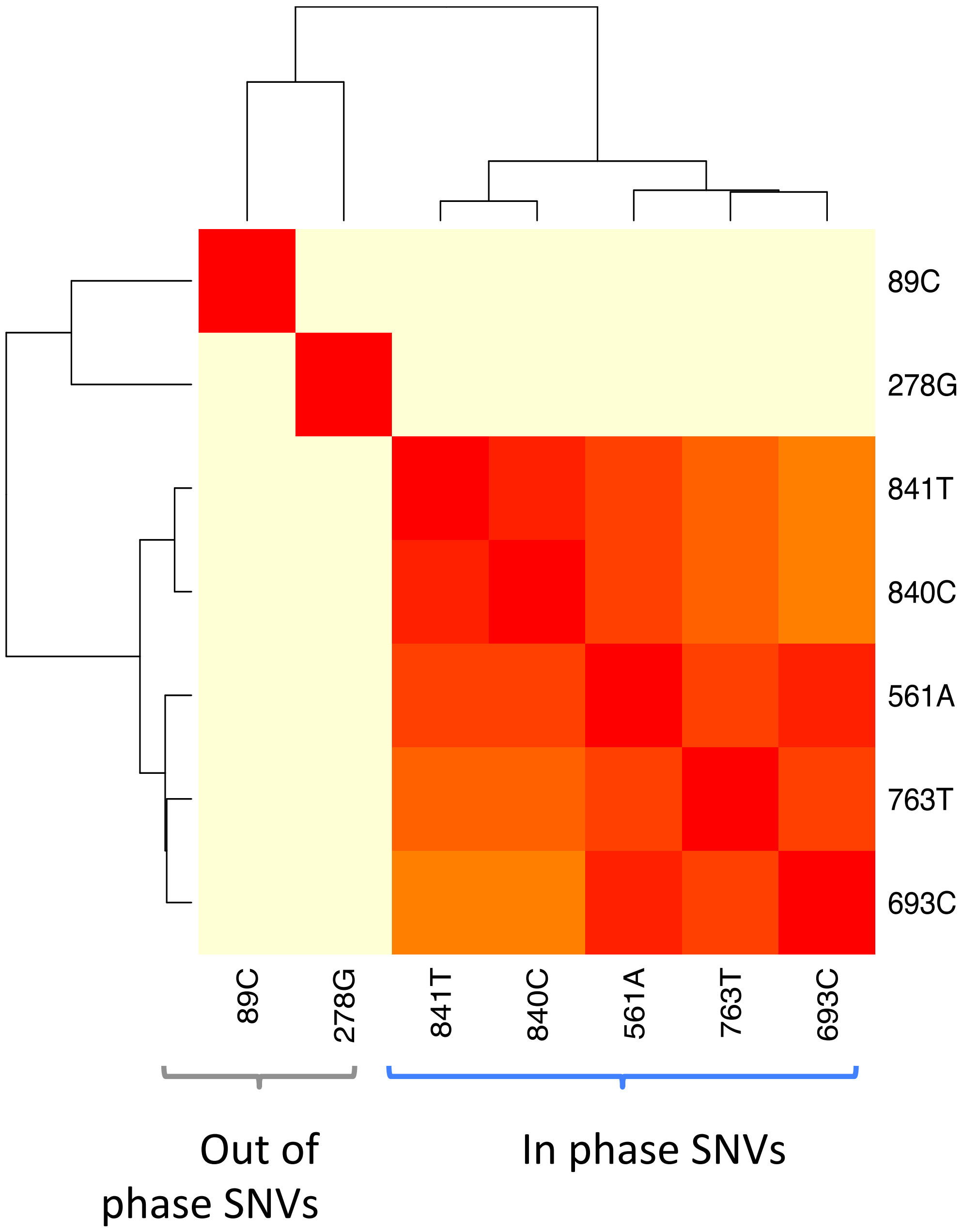

Supplement: Additional file 6: — IRMA figure output. The read percentage chart, coverage/variant diagram, and phasing heat map produced by IRMA are displayed and explained. (PDF 166 kb) [file 12864_2016_3030_MOESM6_ESM.pdf]
