# Supplementary material for: Viral deep sequencing needs an adaptive approach: IRMA, the iterative refinement meta-assembler
Source: BMC Genomics. 2016 Sep 5;17(1):708. doi: 10.1186/s12864-016-3030-6 (PMC5011931; doi:10.1186/s12864-016-3030-6)

Figures relates to the H3N2 calibration mixture

ADDITIONAL FILE 7

MP, replicate 2

Viral parent donor 1

Parent donor consensus sequence differences:

G561A

T693C

G763T

T841C

C842T

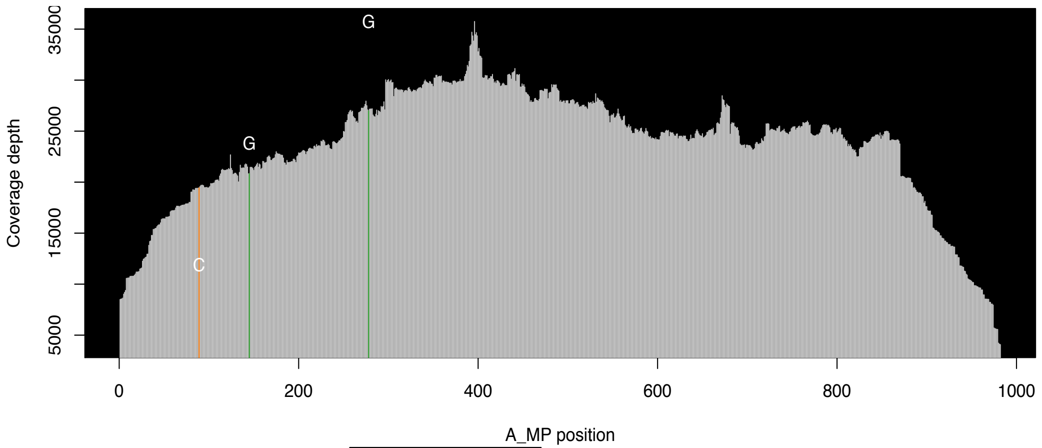

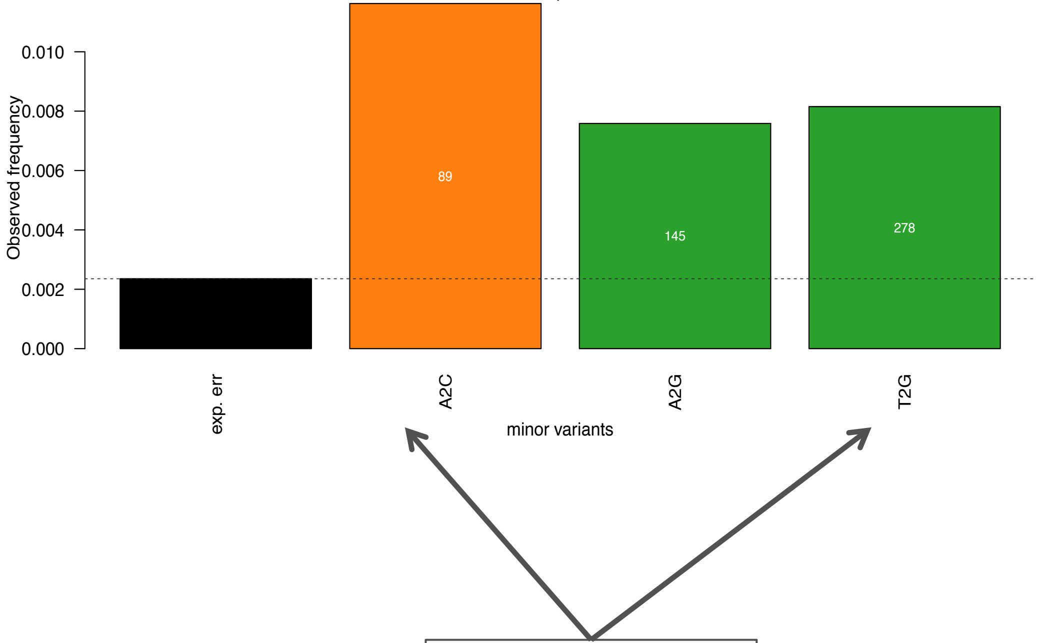

Shared SNVs

MP, replicate 2

Viral parent donor 2

Parent donor consensus sequence differences:

G561A

T693C

G763T

T841C

C842T

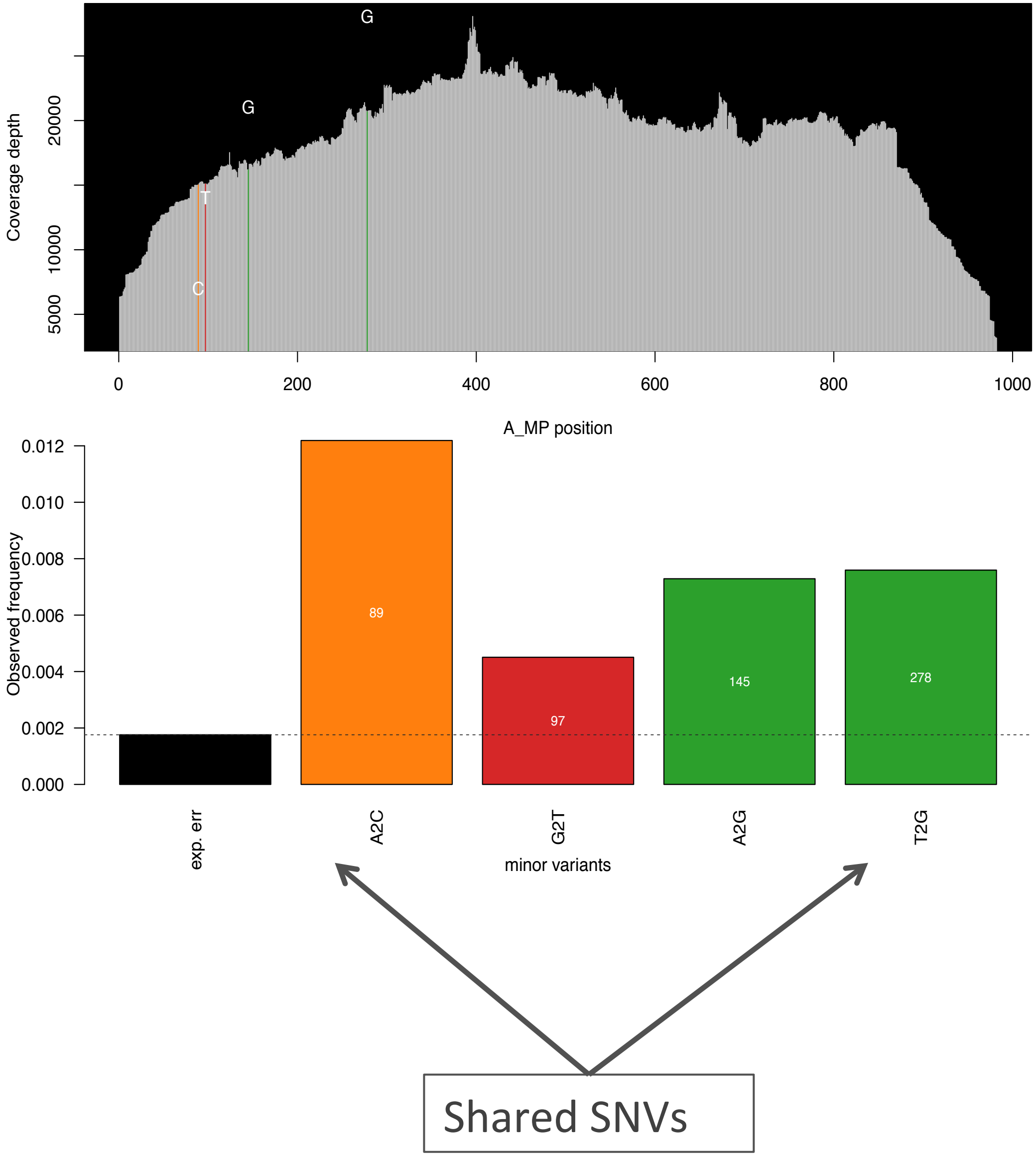

MP, replicate 2

99.5% to 0.5%

SNVs for the minority phase were not called in replicate 2 due to calling criteria and the available statistical support for the observations.

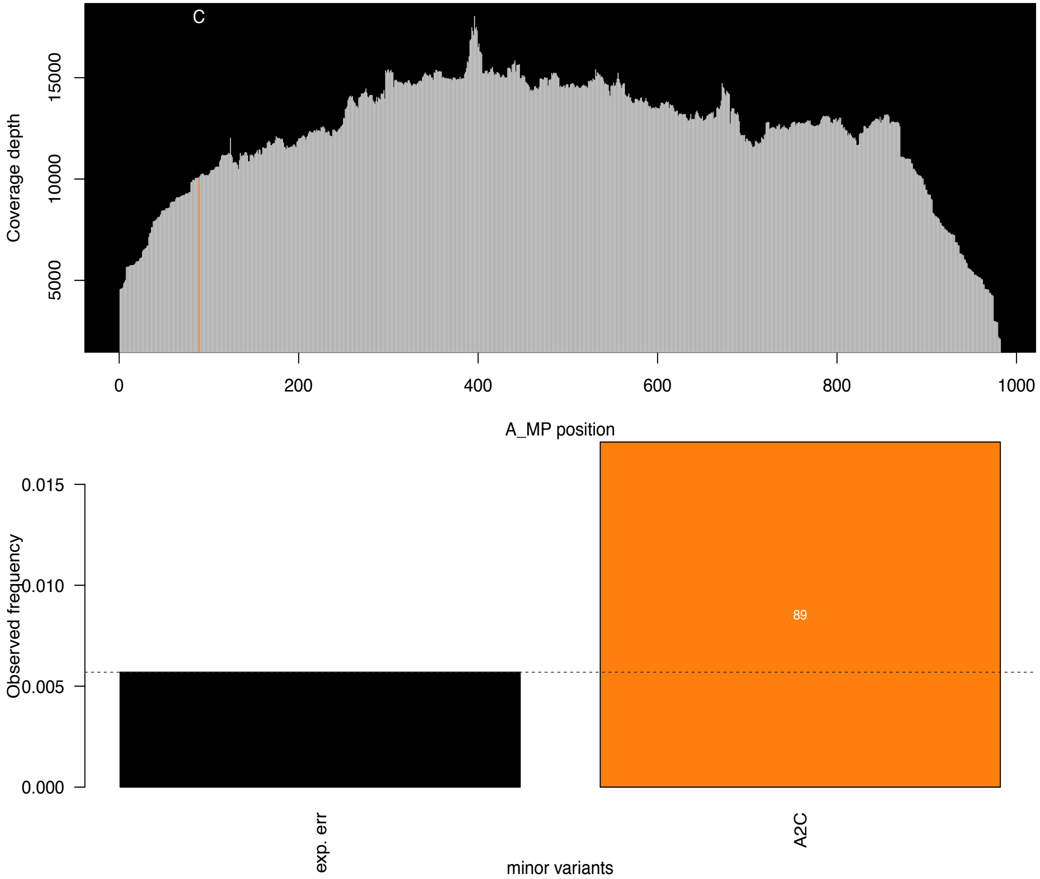

MP, replicate 2

Mix of 99% to 1%

Majority phase: GTGTC
Variant phase: ACTCT

Shared SNVs:
89C
278G

1%

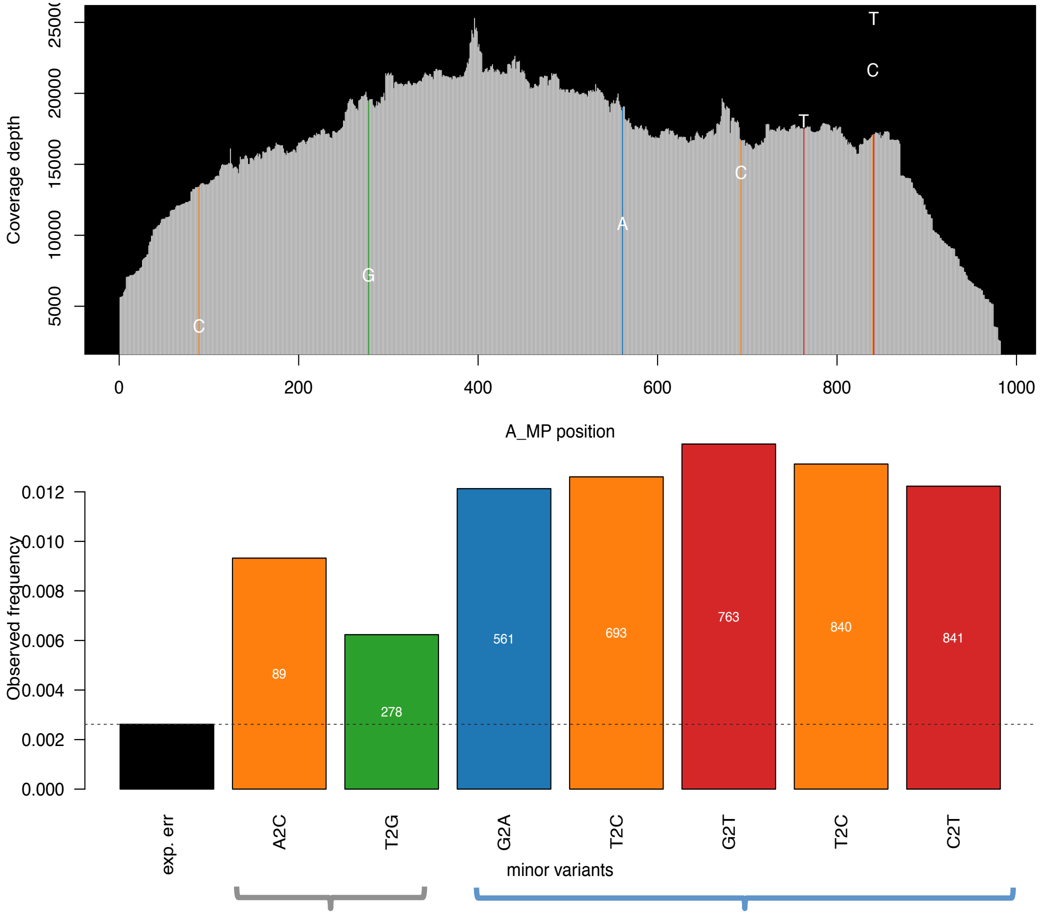

Out of phase SNVs

In phase SNVs

MP, replicate 2

Mix of 99% to 1%

Majority phase: GTGTC
Variant phase: ACTCT

Shared SNVs:
89C
278G

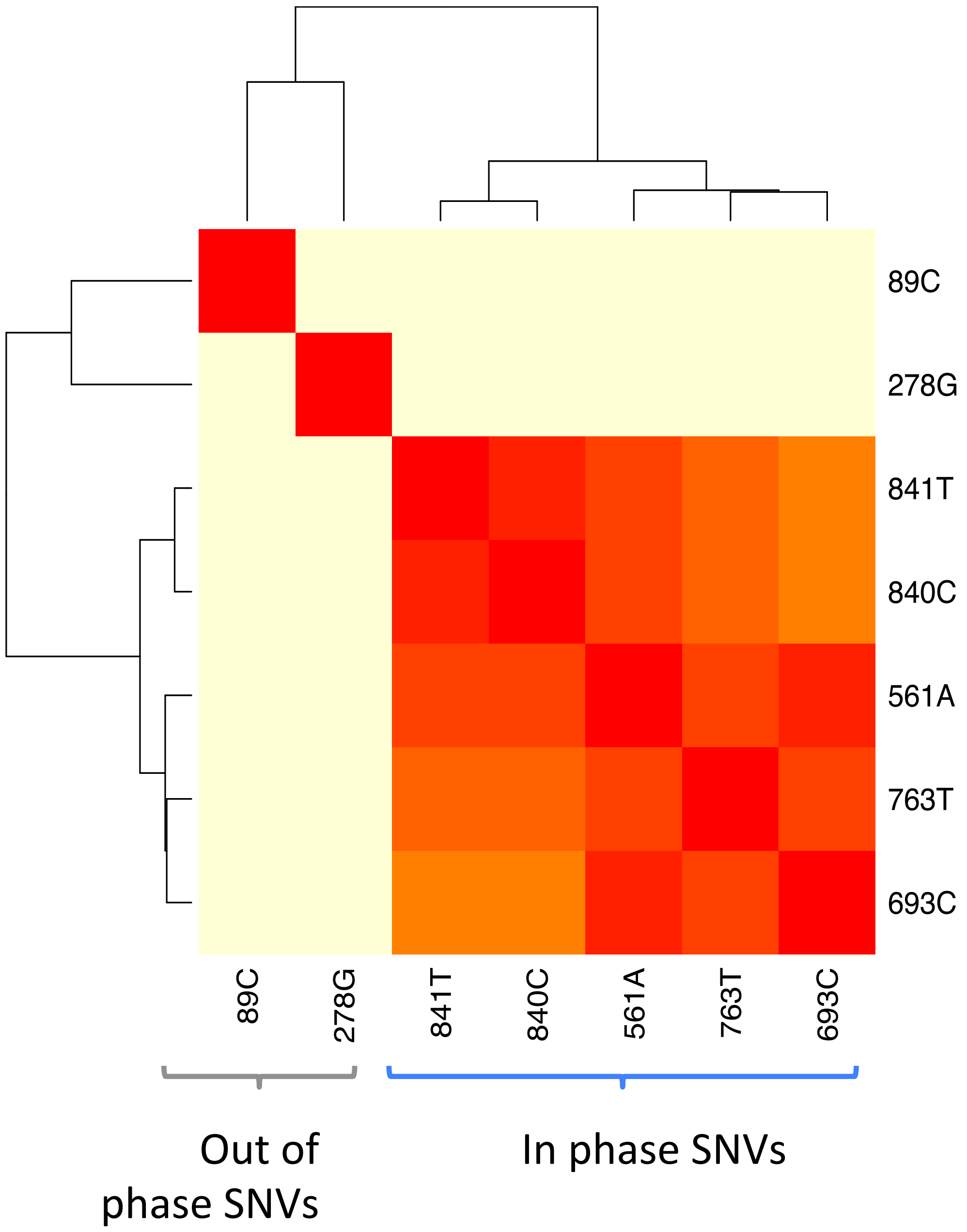

MP, replicate 2

Mix of 98% to 2%

Majority phase: GTGTC

Variant phase: ACTCT

Shared SNVs:

89C

278G

2%

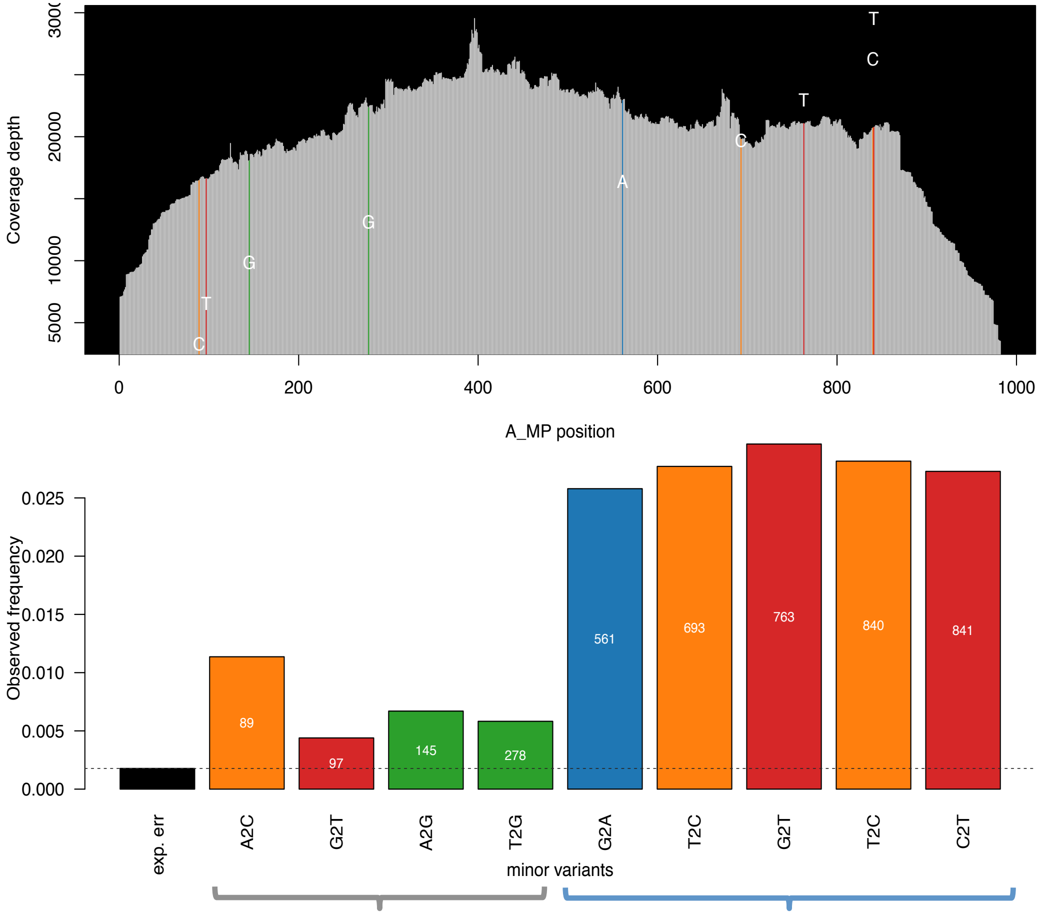

Out of phase SNVs

In phase SNVs

MP, replicate 2

Mix of 98% to 2%

Majority phase: GTGTC

Variant phase: ACTCT

Shared SNVs:

89C

278G

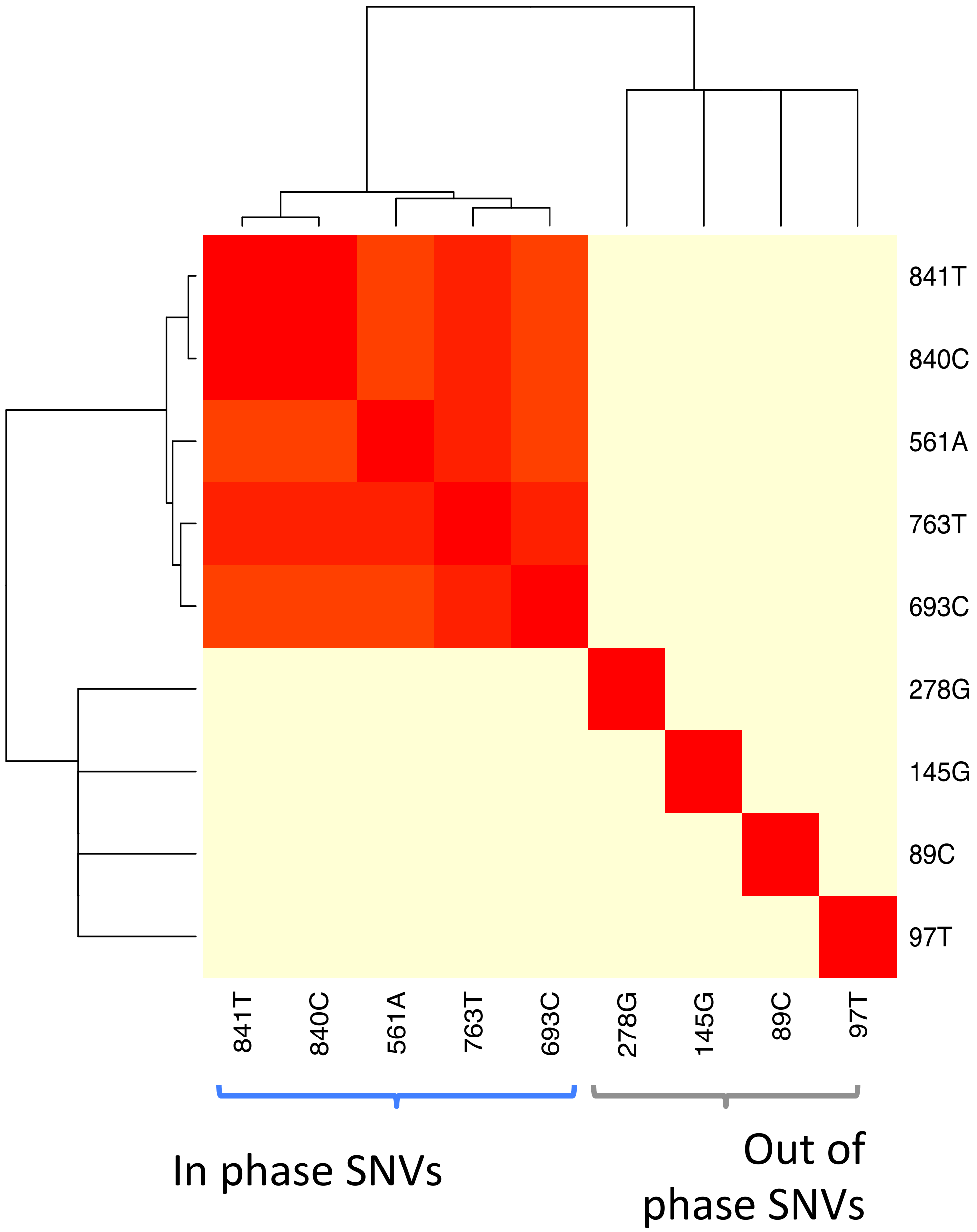

MP, replicate 2

Mix of 95% to 5%

Majority phase: GTGTC
Variant phase: ACTCT

Shared SNVs:
89C
278G

5%

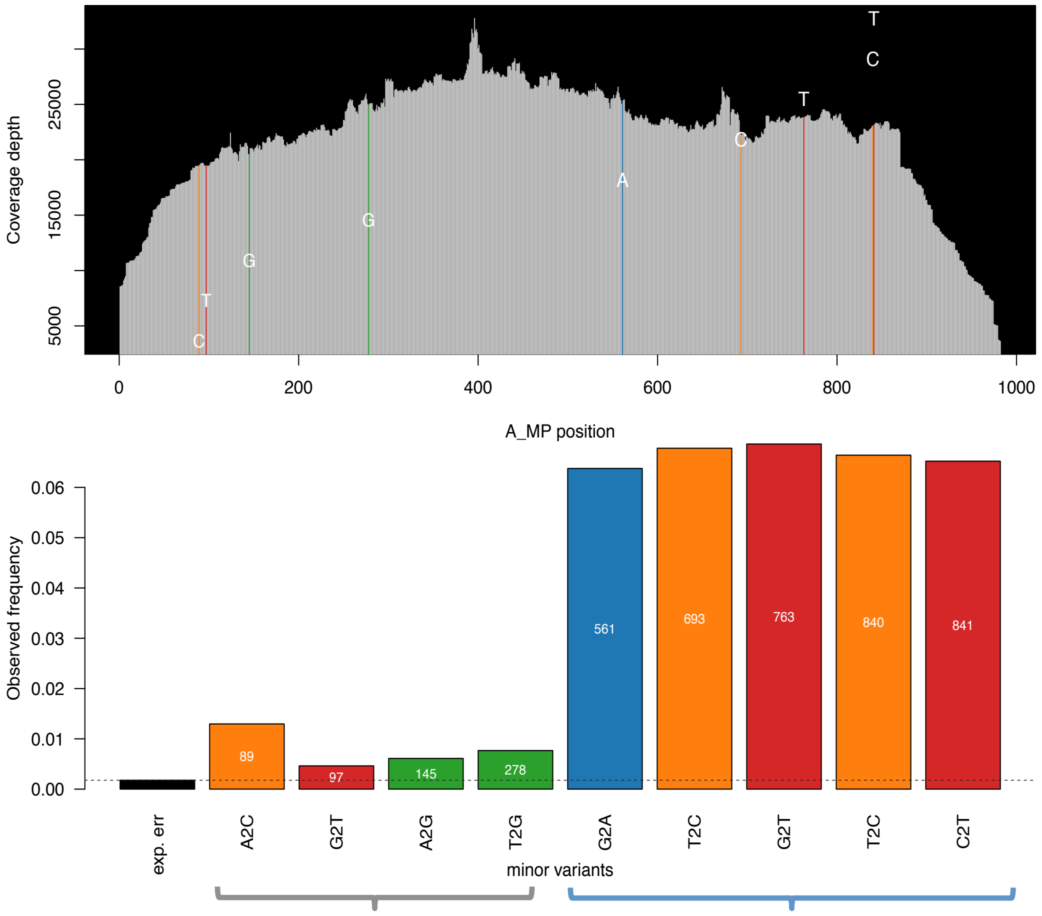

Out of phase SNVs

In phase SNVs

MP, replicate 2

Mix of 95% to 5%

Majority phase: GTGTC

Variant phase: ACTCT

Shared SNVs:

89C

278G

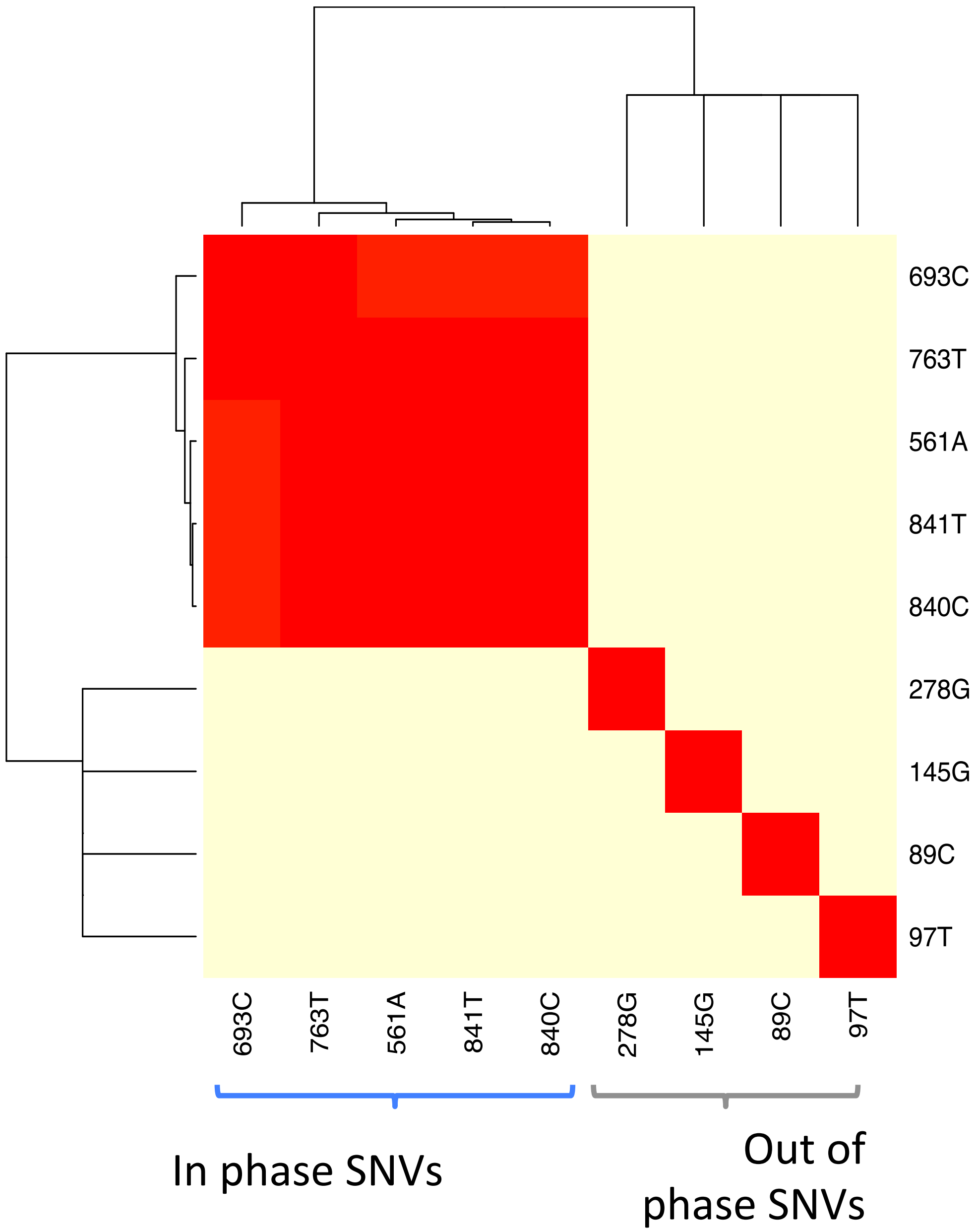

MP, replicate 2

Mix of 90% to 10%

Majority phase: GTGTC

Variant phase: ACTCT

Shared SNVs:

89C

278G

10%

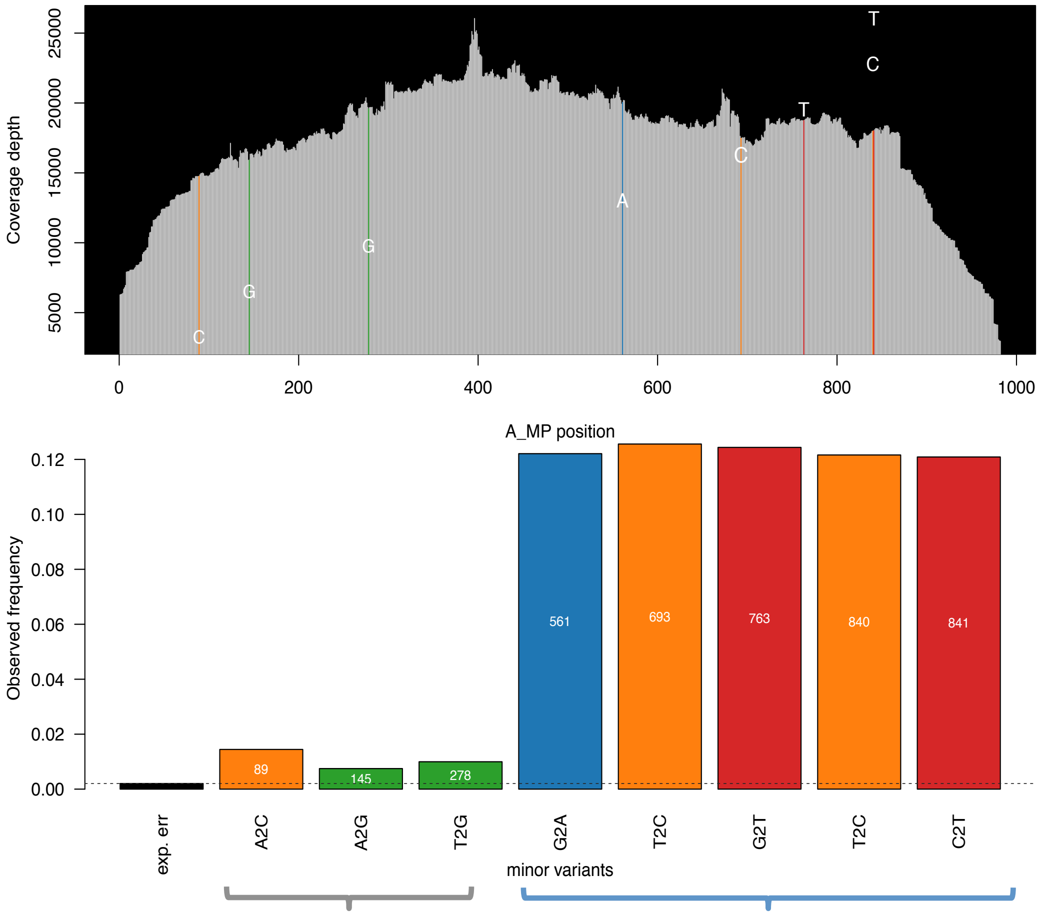

Out of phase SNVs

In phase SNVs

MP, replicate 2

Mix of 90% to 10%

Majority phase: GTGTC
Variant phase: ACTCT

Shared SNVs:
89C
278G

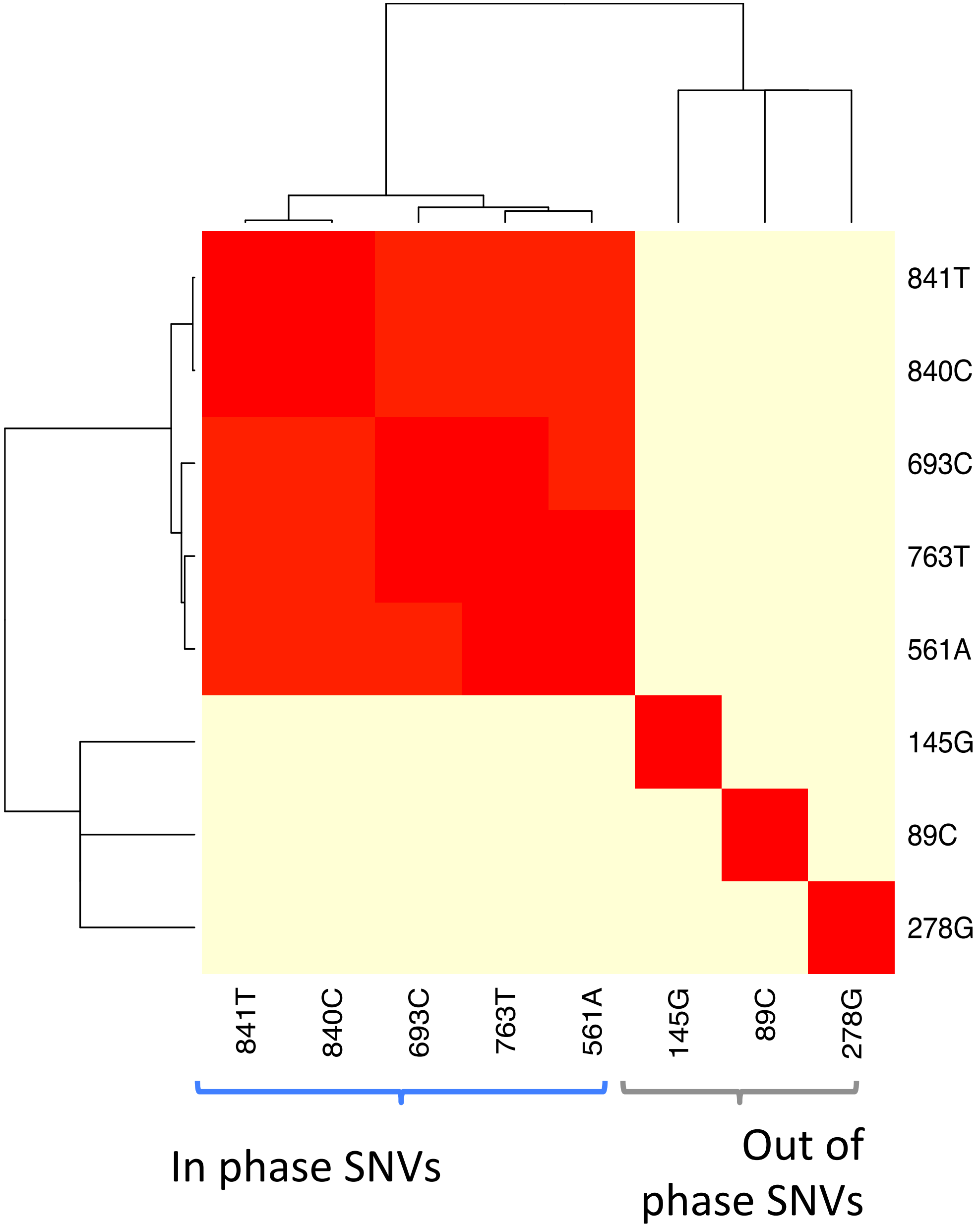

MP, replicate 2

Mix of 80% to 20%

```
Majority phase: GTGTC
Variant phase:  ACTCT
```

```
Shared SNVs:
 89C
278G
```

20%

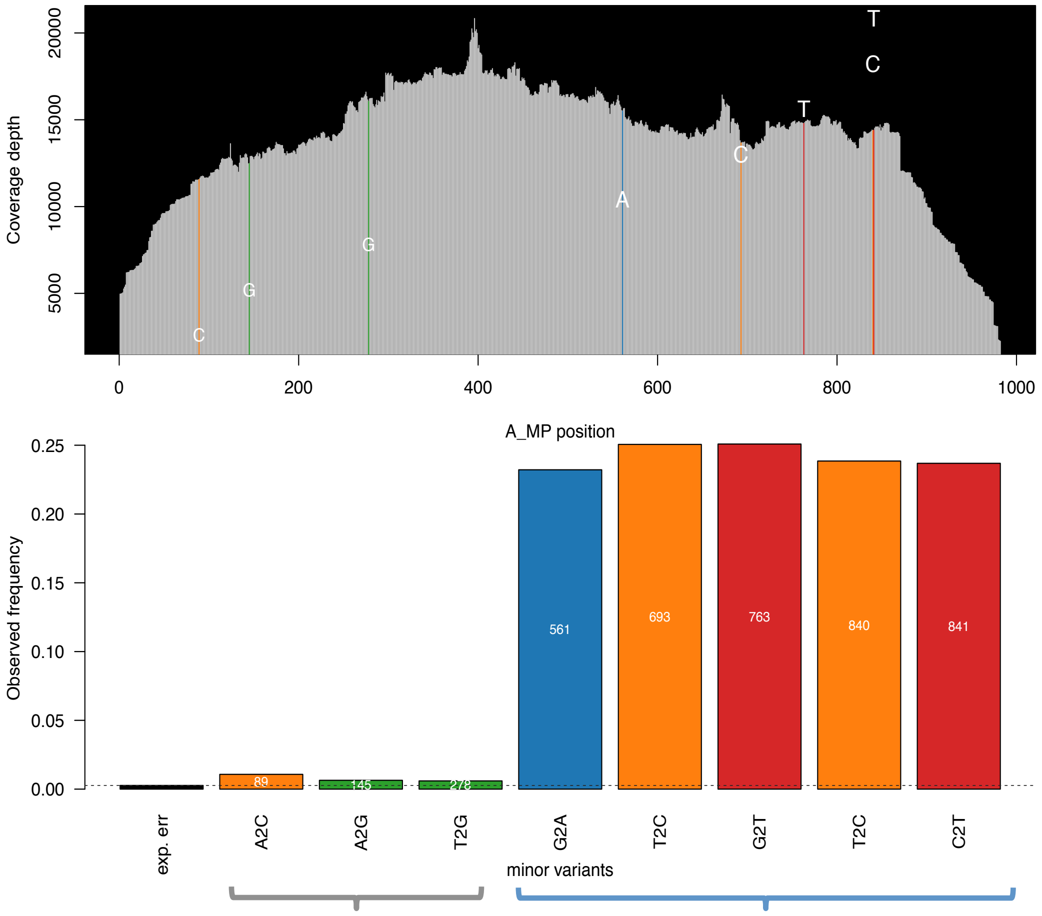

Out of phase SNVs

In phase SNVs

MP, replicate 2

Mix of 80% to 20%

Majority phase: GTGTC
Variant phase: ACTCT

Shared SNVs:
89C
278G

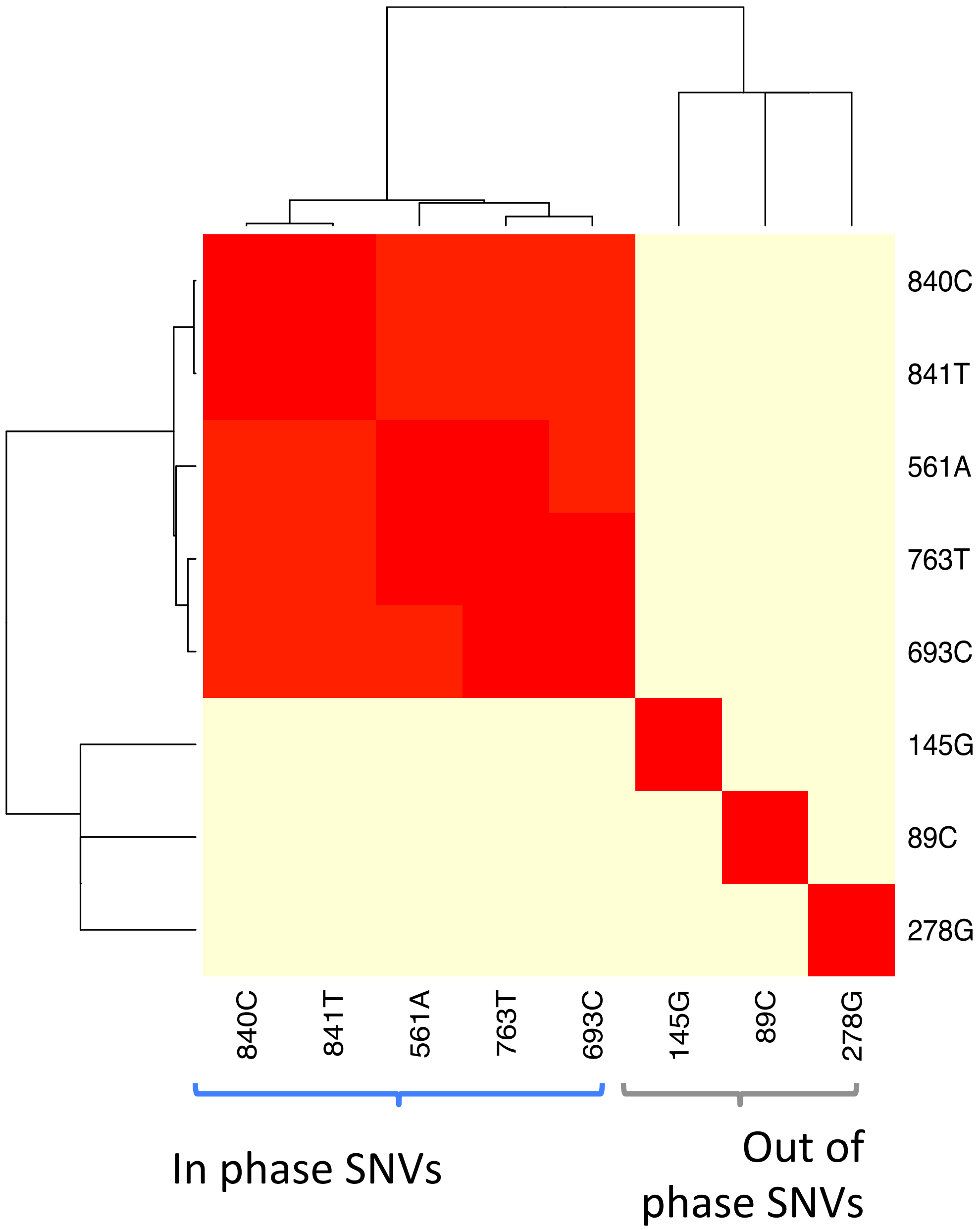

MP, replicate 2

Mix of 50% to 50%

Donor 1 phase: GTGTC

Donor 2 phase: ACTCT

Shared SNVs:

89C

278G

>40%

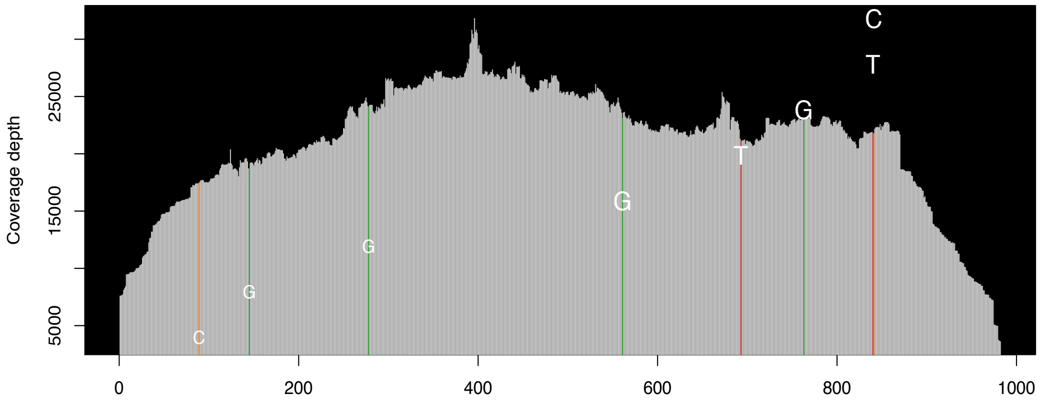

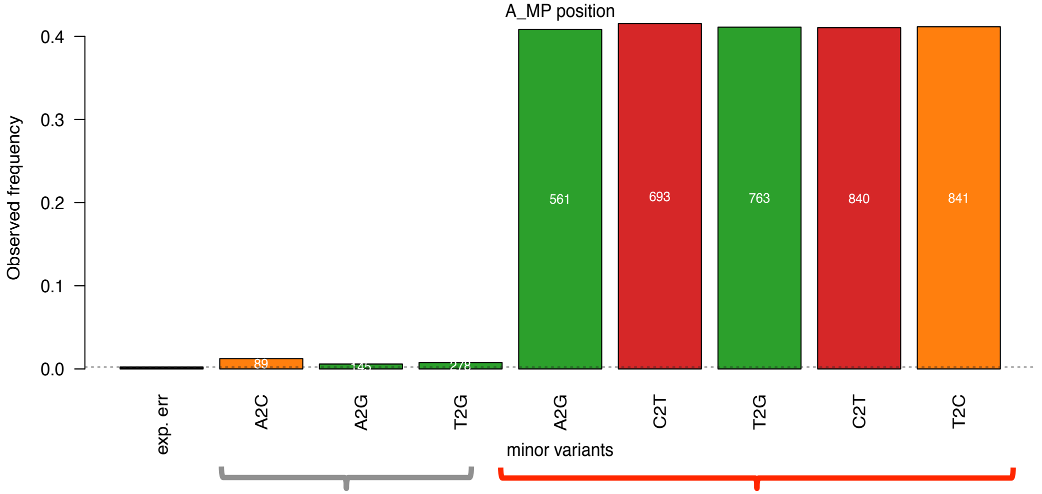

Out of phase SNVs

In phase SNVs

MP, replicate 2

Mix of 50% to 50%

Donor 1 phase: GTGTC

Donor 2 phase: ACTCT

Shared SNVs:

89C

278G

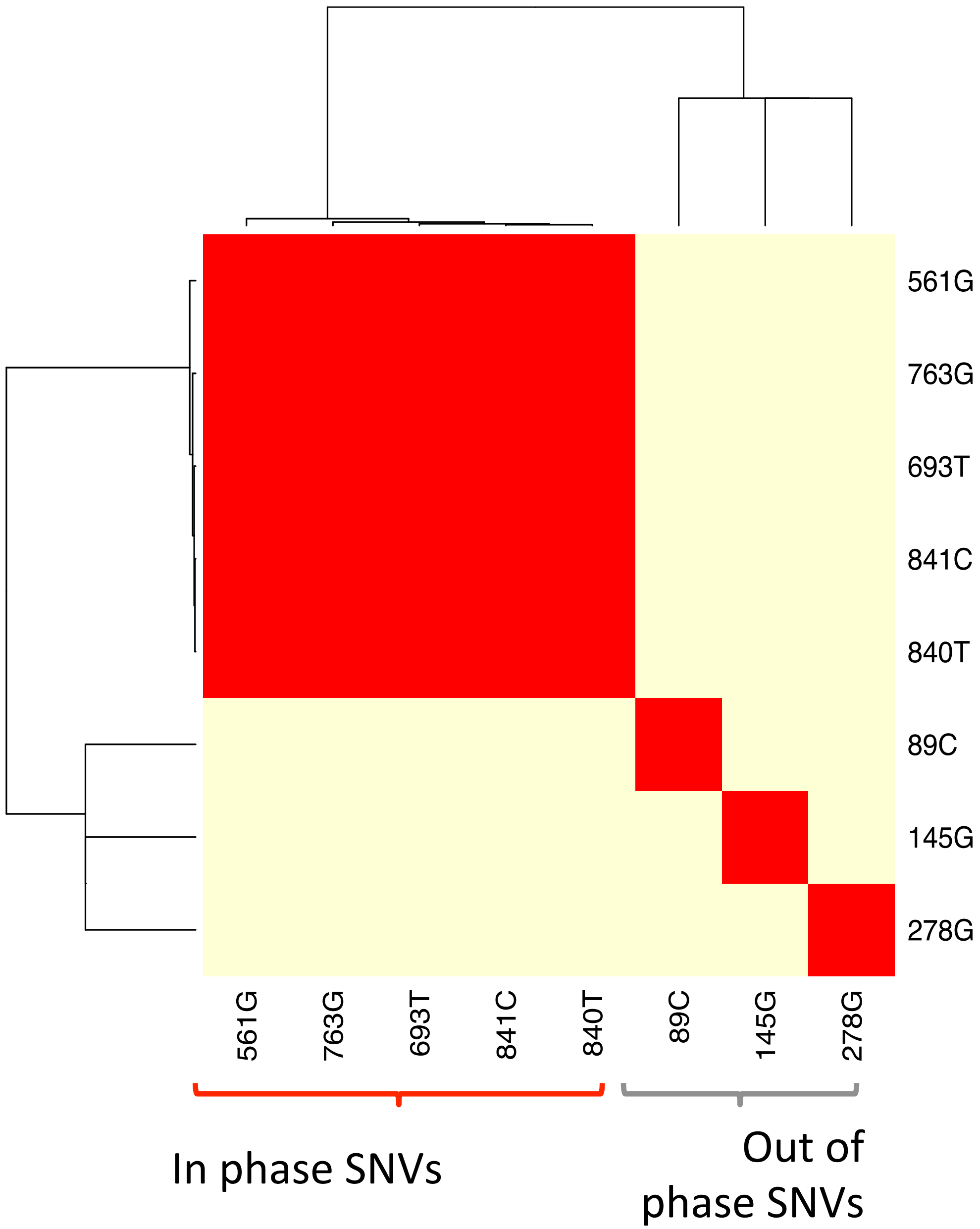

MP, replicate 2

Mix of 25% to 75%

Variant phase: GTGTC

Majority phase: ACTCT

Shared SNVs:

89C

278G

25%

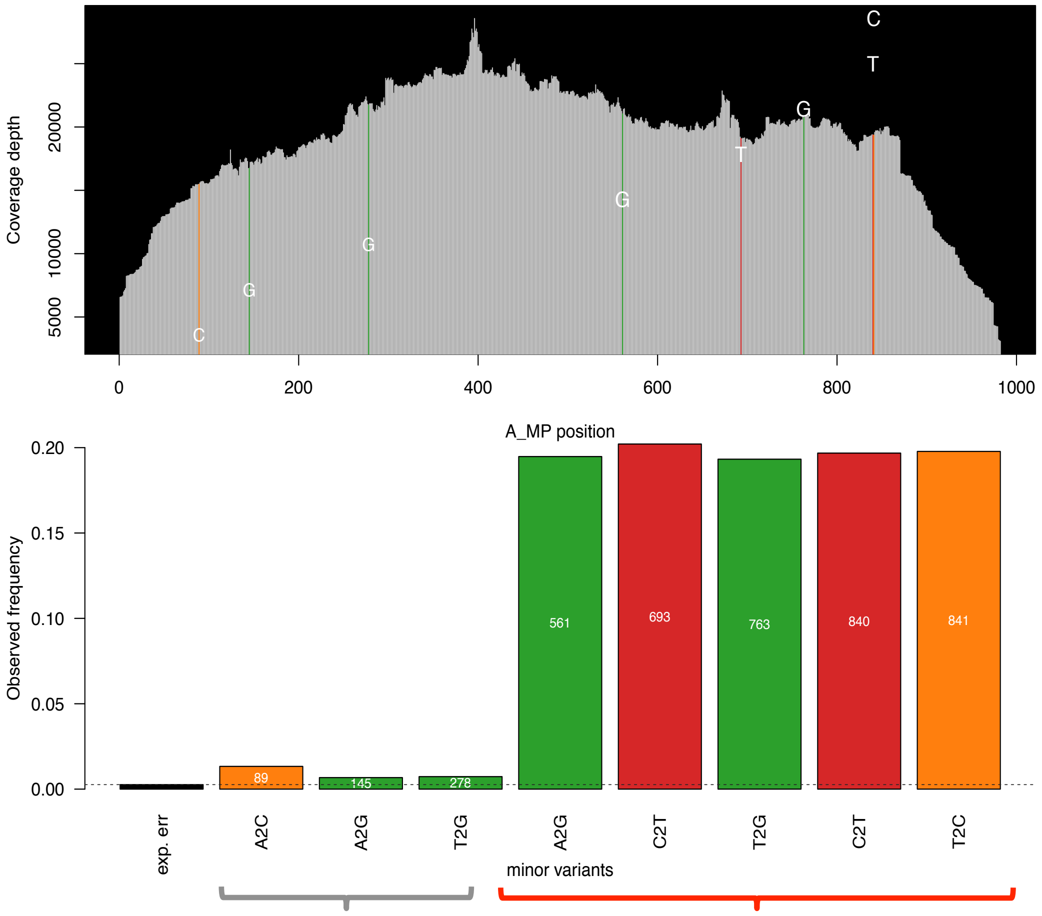

Out of phase SNVs

In phase SNVs

MP, replicate 2

Mix of 25% to 75%

Variant phase: GTGTC
Majority phase: ACTCT

Shared SNVs:
89C
278G

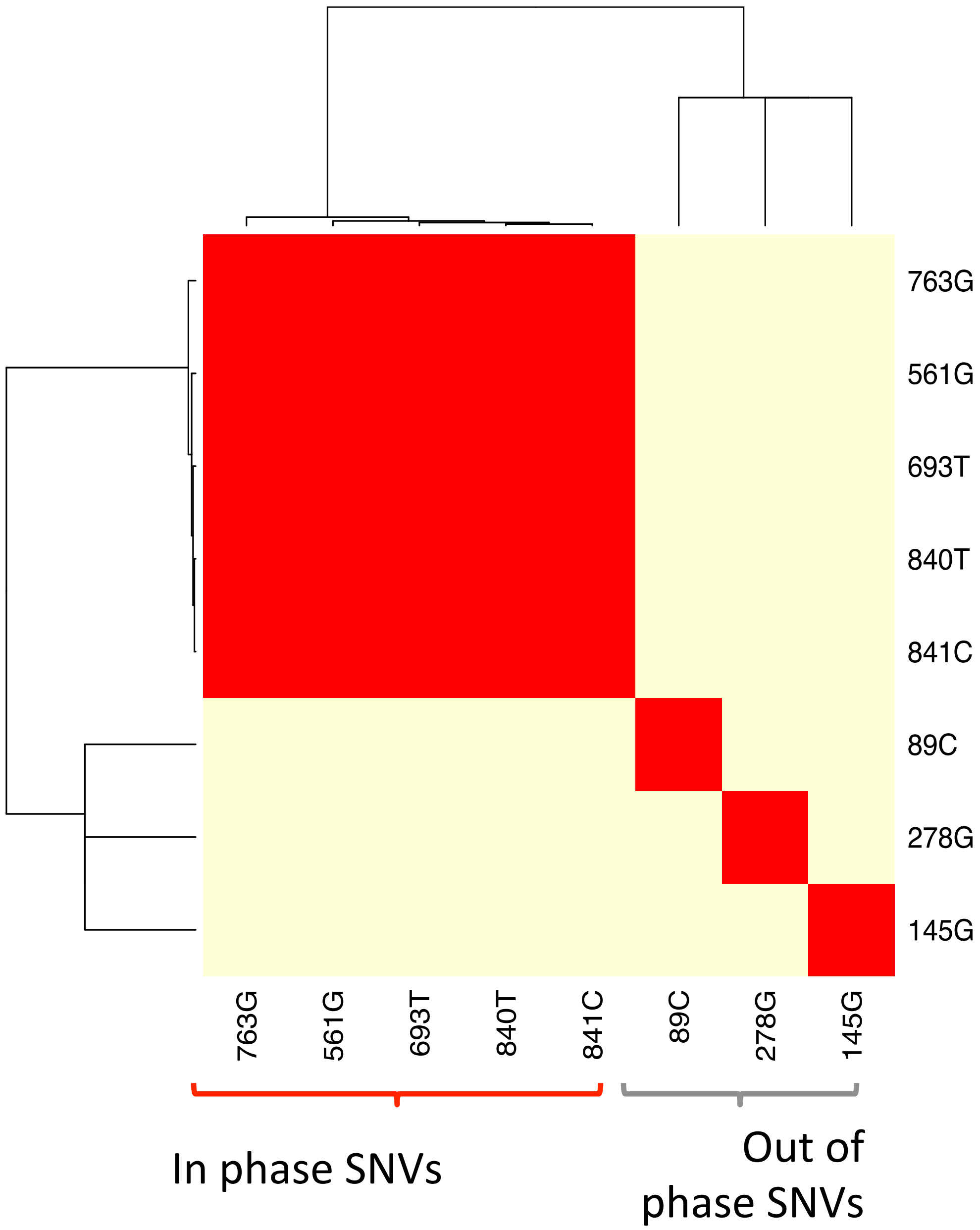

Supplement: Additional file 7: — Phasing information for artificially mixed H3N2 viruses. For each mixture of replicate 2, heat maps show the matrix protein’s minority phases. The minimum variant calling frequency was relaxed to 0.25 % for these mixtures. (PDF 569 kb) [file 12864_2016_3030_MOESM7_ESM.pdf]
