# Supplementary material for: Viral deep sequencing needs an adaptive approach: IRMA, the iterative refinement meta-assembler
Source: BMC Genomics. 2016 Sep 5;17(1):708. doi: 10.1186/s12864-016-3030-6 (PMC5011931; doi:10.1186/s12864-016-3030-6)

Transitive inference of phased singled nucleotide variants

ADDITIONAL FILE 8

NA N2, replicate 2

Mix of 90% to 10%

Known parent donor virus consensus differences:

C 51T
A 88G
T 230C
T 303C
G 321A
C 399T
G 622A
G 651A
A 800C
T 897C
A1080G
G1089A
G1138A
A1176G
C1212T
G1272A
C1396T

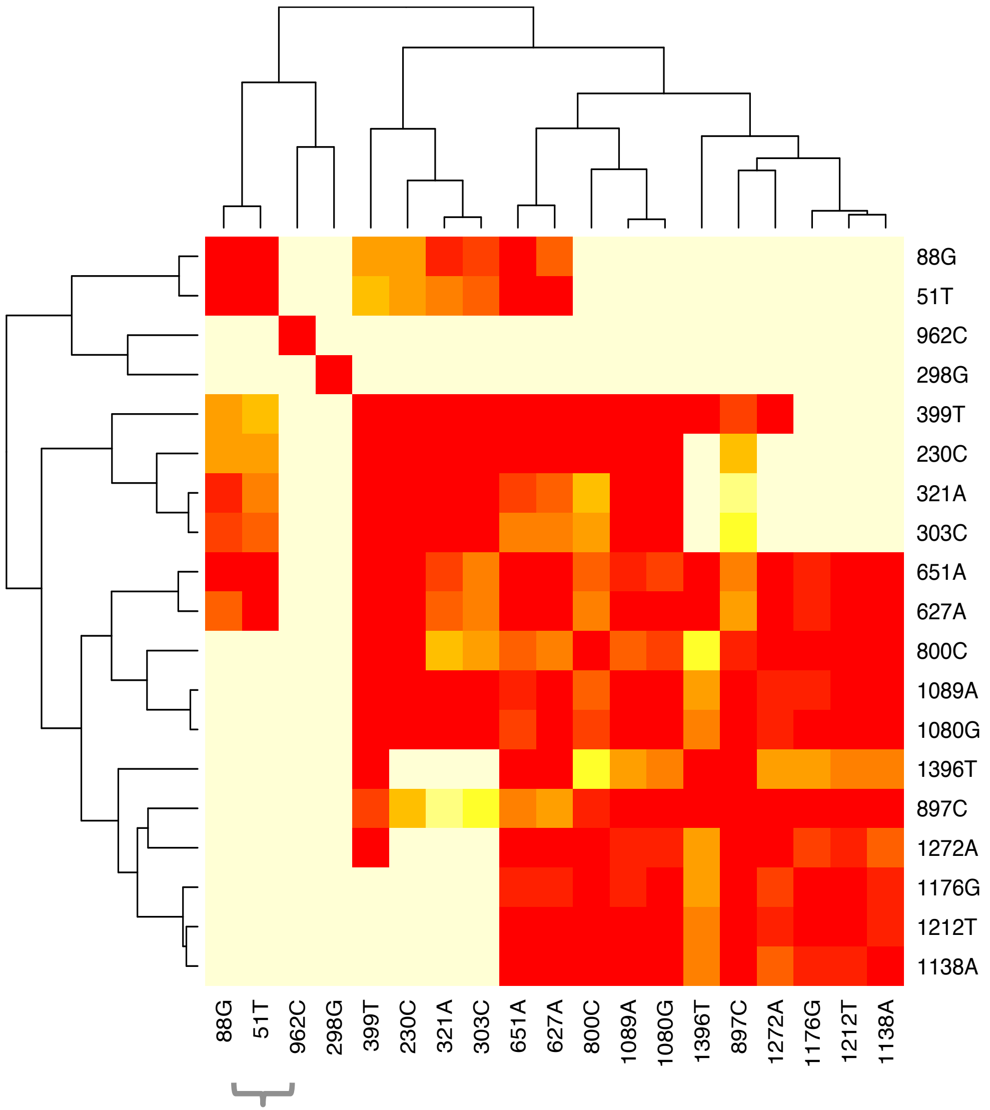

Out of phase SNVs

NA N2, replicate 2

Mix of 90% to 10%

Notice: ***51T*** *is too far away from* ***1396T*** *to be linked due to short read lengths.*

If **51T** and **651A** are linked,
and **651A** and **1396T** are linked,
we might infer, transitively, that
51T and **1396T** are linked.

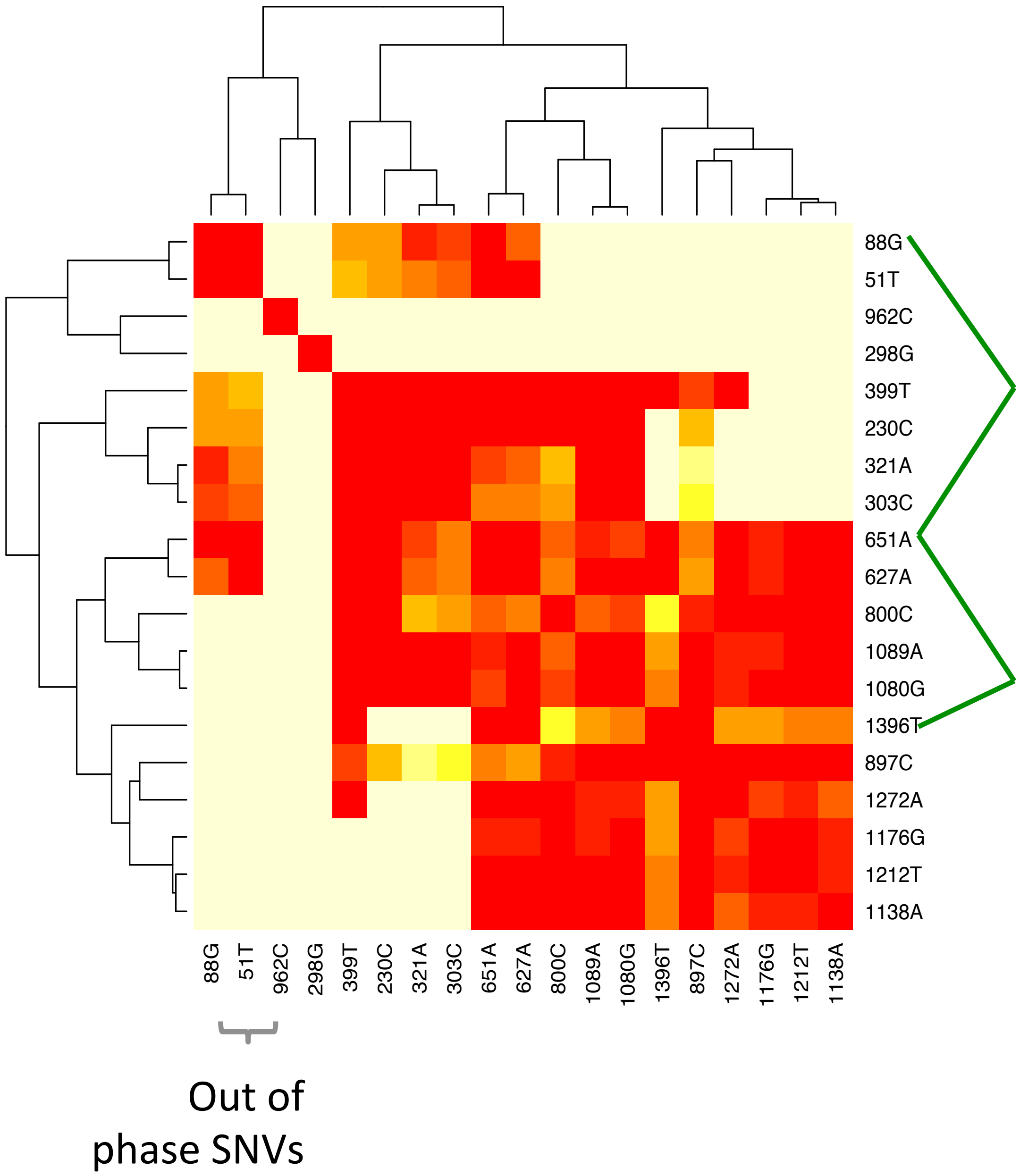

NA N2, replicate 2

Mix of 90% to 10%

Fully linked clusters:

A = {51T, 88G, 230G, 303C, 321A, 399G, **627A**, **651A** }

B = {**627A**, **651A**, 800C, 897C, 1080G, 1089A, 1138A, 1176G, 1212T, 1272A}

Since A and B share SNVs 627A and 651A, we might combine them:

A+B = {230G, 303C, 321A, 399G, **627A**, **651A**, 800C, 897C, 1080G, 1089A, 1138A, 1176G, 1212T, 1272A }

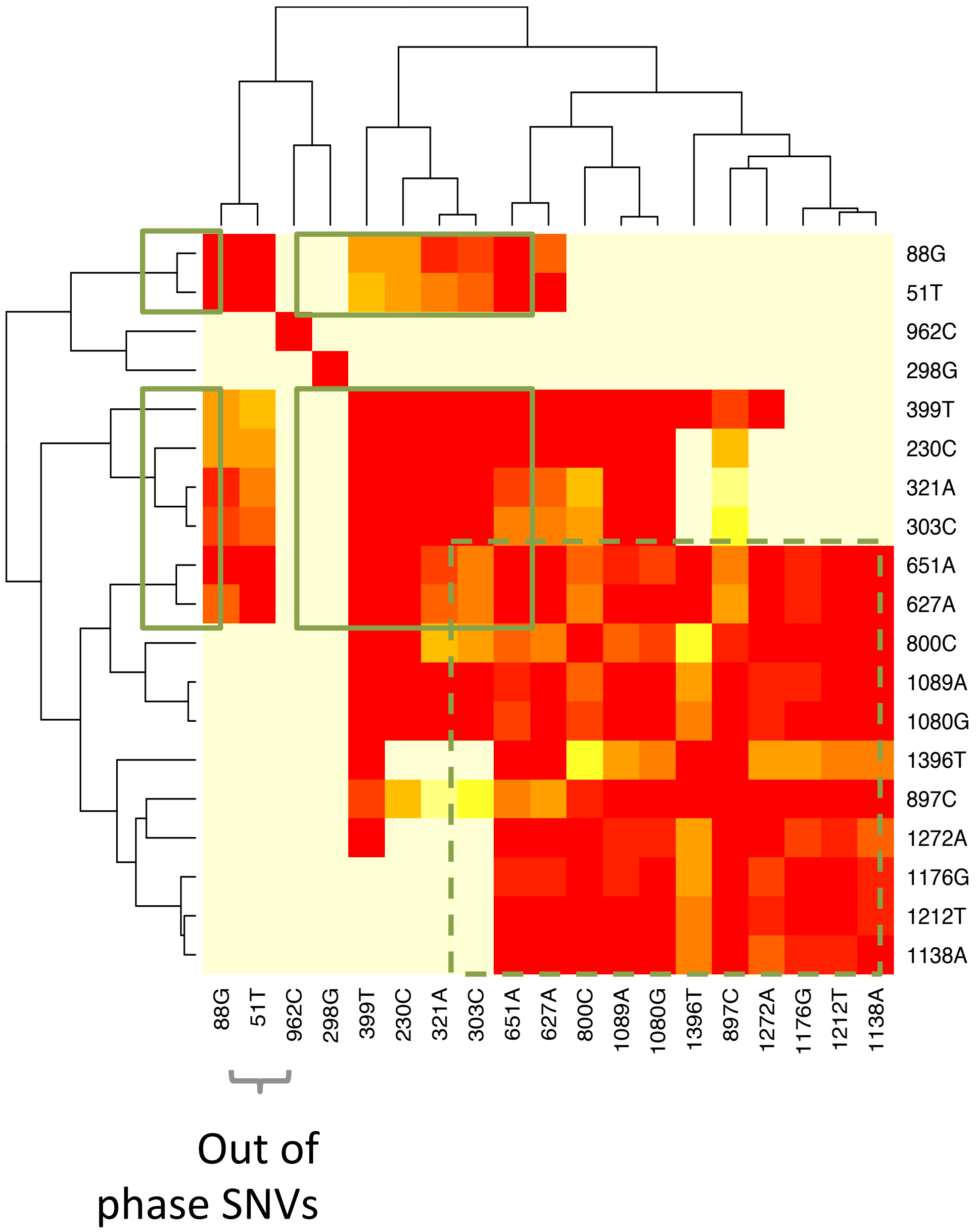

Supplement: Additional file 8: — Inferring phased clusters via the transitive property. Neuraminidase phased variants for the 9:1 mixture of replicate 2 are shown. (PDF 141 kb) [file 12864_2016_3030_MOESM8_ESM.pdf]
