# Supplementary material for: Viral deep sequencing needs an adaptive approach: IRMA, the iterative refinement meta-assembler
Source: BMC Genomics. 2016 Sep 5;17(1):708. doi: 10.1186/s12864-016-3030-6 (PMC5011931; doi:10.1186/s12864-016-3030-6)
Supplement: Additional file 10: — Supplementary methods and formulas used for the variant phasing. (PDF 113 kb) [file 12864_2016_3030_MOESM10_ESM.pdf]

Additional file 10: supplementary methods

Calculation of allele statistics and heuristics

Consider an allele $a \in \mathbb{A} = \{\text{A,T,C,G}\}$ at some fixed position p within an assembly of $1 \leq p \leq S$ sites. Let:

$$C_{a,p} \qquad \text{count of allele } a \text{ at position } p \tag{A.1}$$

$$D_p = \sum_{a \in \mathbb{A}} C_{a,p} \qquad \text{coverage depth at position } p \tag{A.2}$$

$$f_{a,p} = \frac{C_{a,p}}{D_p} \qquad \text{observed frequency of allele } a \text{ at position } p \tag{A.3}$$

Suppose also that for each read i at position p and allele a we have quality score q_i such that for $1 \leq i \leq C_{a,p}$ we have:

$$Q_{a,p} = \frac{\sum_i^{C_{a,p}} q_i}{C_{a,p}} \qquad \text{average of allele } a \text{ quality scores } q_i \text{ at } p \tag{A.4}$$

Quality-based expected error is calculated as usual but on the average allele quality score:

$$\hat{e}_{a,p} = \frac{1}{10^{\frac{Q_{a,p}}{10}}} \qquad \text{expected allele-specific error at position } p \tag{A.5}$$

The read-pair overlap disagreement rate $\hat{e}_S$ is calculated over all sites where overlap occurs between read-pairs:

$$\hat{e}_S = \frac{\text{\# disagreeing overlapping read-pair observations}}{\text{\# overlapping read-pair observations}} \qquad \text{assembly-specific error} \tag{A.6}$$

Our computation of the one-sided second-order corrected binomial confidence interval upperbound [40] takes an estimate of error $\hat{e}$ (either $\hat{e}_{a,p}$ or $\hat{e}_S$), a coverage depth D_p corresponding to the variant allele site, and a z score corresponding to the chosen one-sided confidence level (α) of the standard normal distribution [e.g., $P(Z < z) = \alpha$ for some $\alpha \in \{0.90, 0.95, 0.99, 0.999\}$]:

$$\eta = \frac{1}{3}z^2 + \frac{1}{6}, \quad \gamma_1 = \frac{13}{18}z^2 + \frac{17}{18}, \quad \gamma_2 = \frac{1}{18}z^2 + \frac{7}{36}, \quad \text{variance } V \equiv \sigma^2 = \hat{e} - \hat{e}^2$$

$$U(\hat{e}, D_p, z) = \frac{D_p \cdot \hat{e} + \eta}{D_p + 2\eta} + z\frac{\sqrt{V + (\gamma_2 - \gamma_1 V)D_p^{-1}}}{\sqrt{D_p}} \tag{A.7}$$

Statistically significant minor variants must reject the null hypothesis that their observed frequency $f_{a,p}$ can be explained by estimates of error $\hat{e}$. Therefore, observed frequencies must exceed the interval upperbound at some confidence level α with corresponding score z_α:

$$f_{a,p} > U(\hat{e}, D_p, z_\alpha) \tag{A.8}$$

Our heuristic for confidence not machine error is calculated as:

$$\begin{aligned} \hat{f}_{a,p} &= \max\{0, f_{a,p} - \hat{e}_{a,p}\} && \text{estimated frequency} \\ m_{a,p} &= \frac{\hat{f}_{a,p}}{f_{a,p}} && \text{confidence not sequencer error} \end{aligned} \tag{A.9}$$

Automatic heuristic adjustment is calculated over the set of non-consensus alleles A that have "zero confidence":

$$\begin{aligned} A_0 &= \{(a,p) \in A \mid m_{a,p} = 0\} && \text{subset with zero confidence} \\ F_{\min} &= \text{user defined in: } [0,0.5] && \text{minimum frequency heuristic} \\ F_{\text{auto}} &= \max\{F_{\min}, \arg\max_{a,p \,\in\, A_0} f_{a,p}\} && \text{automatic frequency heuristic} \end{aligned} \tag{A.10}$$

Association measures for phased minor variants

Consider an assembly of S sites with coverage depths denoted D_x for $1 \leq x \leq S$. The "joint coverage depth" or number of reads with defined calls for any fixed pairs of sites x, y we denote as $D_{x,y}$ with the following properties:

$$D_{x,x} = D_x$$

$$D_{x,y} = 0 \iff x, y \text{ never share a read}$$

$$D_{x,y} \leq \min D_x, D_y$$

Next consider an allele $B \in \{A, G, C, T\}$ at site x. Let $C_x(B|s = x)$ be the count of allele B at x given the occurrence of site x. This could also just be written $C_x(B)$. One can further define the count of allele B at x given the co-occurrences of multiple sites x, y as $C_x(B|s_1 = x \;\&\; s_2 = y)$. The frequency definitions follow:

$$F_x(B|s = x) = \frac{C_x(B)}{D_x} \tag{A.11}$$

$$F_x(B|s_1 = x \;\&\; s_2 = y) = \frac{C_x(B|s_1 = x \;\&\; s_2 = y)}{D_{x,y}} \tag{A.12}$$

$$F_x^{(min)}(B) = \min\{F_x(B|s = x), F_x(B|s_1 = x \;\&\; s_2 = y)\} \tag{A.13}$$

where $F_x^{(min)}(B)$ can be considered a conservative estimate of individual site allele frequency. The corollary definition of $F_x^{(max)}(B)$ is the relaxed estimate. Joint frequency in terms of a short-read assembly is defined as:

$$F_{x,y}(B_1, B_2) = \frac{C_{x,y}(B_1, B_2)}{D_{x,y}} \tag{A.14}$$

where B_1 and B_2 are alleles at x and y respectively and $C_{x,y}(B_1, B_2)$, or more explicitly $C_{x,y}(B_1, B_2 | s_1 = x \ \& \ s_2 = y)$, is the count of the alleles' co-occurrence given both sites occur on the read together.

From the study of di-nucleotide signatures within various genomes [42], di-nucleotide "enrichment" may be defined as the ratio of the joint probability for two bases (actual co-occurrence) over the product of the individual probabilities for each base (random expectation of their co-occurrence). The elevated co-occurrence of phased minor variants ought to be detectable using similar mathematical constructions. However, such an enrichment measure is not normalized to give us a proper distance. Therefore, we may re-frame the frequencies/probabilities in terms of association measures commonly used in text mining literature [20]. Such association measure distances are easier to use and interpret for visualization. First, we introduce a slightly modified Jaccard distance [21]:

$$J_{x,y}(B_1, B_2) = 1 - \frac{F_{x,y}(B_1, B_2)}{F_x^{(max)}(B_1) + F_y^{(max)}(B_2) - F_{x,y}(B_1, B_2)} \tag{A.15}$$

a slightly modified, non-log mutual dependency distance [20]:

$$M_{x,y}(B_1, B_2) = 1 - \frac{F_{x,y}(B_1, B_2)^2}{F_x^{(max)}(B_1) \cdot F_y^{(max)}(B_2)} \tag{A.16}$$

and our own experimental association distance:

$$E_{x,y}(B_1, B_2) = 1 - \frac{F_{x,y}(B_1, B_2) \cdot \min\{F_x^{(min)}(B_1)\, F_y^{(min)}(B_2)\}}{F_x^{(max)}(B_1) \cdot F_y^{(max)}(B_2)} \tag{A.17}$$

The use of minimums and maximums ensure conservative estimates of association. Additionally, we show what would occur if one were to convert the joint probability to a distance for minority variants:

$$1 - 2 \cdot F_{x,y}(B_1, B_2) \tag{A.18}$$
